# Expert Judgment Supporting a Bayesian Network to Model the Survival of Pancreatic Cancer Patients †

**DOI:** 10.3390/cancers17020301

**Published:** 2025-01-17

**Authors:** Erica Secchettin, Salvatore Paiella, Danila Azzolina, Fabio Casciani, Roberto Salvia, Giuseppe Malleo, Dario Gregori

**Affiliations:** 1 University of Verona, 37134 Verona, Italy; salvatore.paiella@univr.it (S.P.); roberto.salvia@univr.it (R.S.); giuseppe.malleo@univr.it (G.M.); 2 Department of Surgery, Dentistry, Paediatrics and Gynecology, University of Verona, 37134 Verona, Italy; 3 Pancreatic Surgery Unit, Department of Surgery, Dentistry, Paediatrics and Gynecology, University of Verona, 37134 Verona, Italy; fabio.casciani@univr.it; 4 Department of Environmental and Preventive Science, University of Ferrara, 44121 Ferrara, Italy; danila.azzolina@ubep.unipd.it; 5 Pancreatic Surgery Unit, Department of Engineering for Innovation Medicine (DIMI), University of Verona, 37134 Verona, Italy; 6 Unit of Biostatistics, Epidemiology and Public Health, Department of Cardiac, Thoracic and Vascular Sciences, University of Padova, 35122 Padova, Italy; dario.gregori@unipd.it

**Keywords:** Bayesian network, prior elicitation, SHELF, pancreatic cancer, prognosis

## Abstract

**Purpose**: Pancreatic cancer is known for its poor prognosis. The most effective treatment combines surgery with peri-operative chemotherapy. Current prognostic tools are designed to predict patient outcomes and inform treatment decisions based on collected data. Bayesian networks (BNs) can integrate objective data with subjective clinical insights, such as expert opinions, or they can be independently based on either element. This pilot study is one of the first efforts to incorporate expert opinions into a prognostic model using a Bayesian framework. **Methods**: A clinical hybrid BN was selected to model the long-term overall survival of pancreatic cancer patients. The SHELF expert judgment method was employed to enhance the BN’s effectiveness. This approach involved a two-phase protocol: an initial single-center pilot phase followed by a definitive international phase. **Results**: Experts generally agreed on the distribution shape among the 12 clinically relevant predictive variables identified for the BN. However, discrepancies were noted in the tumor size, age, and ASA score nodes. With regard to expert concordance for each node, tumor size, and ASA score exhibited absolute concordance, indicating a strong consensus among experts. Ca19.9 values and resectability status showed high concordance, reflecting a solid agreement among the experts. The remaining nodes showed acceptable concordance. **Conclusions***:* This project introduces a novel clinical hybrid Bayesian network (BN) that incorporates expert elicitation and clinical variables present at diagnosis to model the survival of pancreatic cancer patients. This model aims to provide research-based evidence for more reliable prognosis predictions and improved decision-making, addressing the limitations of existing survival prediction models. A validation process will be essential to evaluate the model’s performance and clinical applicability.

## Simple Summary

A novel hybrid Bayesian network was created to predict pancreatic cancer survival, incorporating experts’ opinions and best literature knowledge using the SHELF method. The hybrid Bayesian network model was developed using twelve clinically validated prognostic variables already available at diagnosis of pancreatic cancer. It aims at overcoming the limit of the lack of threshold values or categories for most clinical variables currently used to predict the survival of pancreatic cancer patients. A validation phase will validate its clinical applicability and reliability.

## 1. Introduction

Pancreatic cancer is projected to become the second leading cause of cancer-related death [1]. The only chance for long-term survival in pancreatic cancer patients lies in the combination of chemotherapy and surgical resection, which is achievable in only 20% of cases. Prediction models focused on time-to-event outcomes after pancreatectomy—such as disease-free survival and overall survival—are essential for guiding treatment decisions and providing patient counseling. Current survival prediction tools primarily rely on inferential statistics and traditional regression techniques, such as the Cox proportional hazards model. However, these methods often do not capture the dynamic nature of the disease and the complexities of care processes. The literature suggests that advanced predictive models, which can incorporate perturbing events (like treatment complications) and time-dependent information, may enhance the predictive accuracy of survival models [2,3,4].

A Bayesian network (BN) implements Bayesian inference for conditional probabilities, allowing for the inclusion of expert opinions when data are limited or for a mixture of both data and opinions when available [5,6,7,8]. In a Bayesian framework, the “prior” represents the initial knowledge about the probability of an outcome and incorporating expert input can stabilize predictions when data are scarce or uncertain.

Thus, it is appropriate to derive priors (so-called “informative priors”) in BN to compute posteriors adequately at the time of including any patient data for prediction. This process avoids biased estimation or difficult posterior computations (so-called “improper posteriors”) [9]. Using “non-informative priors” may complicate model selection and hypothesis testing [10] in a clinical scenario. Therefore, it is essential to assign each node in the BN an informative prior and to connect the nodes using Bayes’ theorem to create a robust model [9].

In pancreatic cancer care, many baseline variables guiding prognostic predictions (serum Ca 19-9 levels, tumor size, or tumor site) do not have a unique threshold or a definite category for reference. Parametrization of “informative priors” may be performed by converting clinical expertise and the best available literature into numerical values through the expert elicitation process, integrating these values into the BN. Expert elicitation is a well-established method for gathering and synthesizing unbiased expert judgments, providing valuable quantitative information when data lack a clear threshold or strong reference points in survival risk models [11]. This approach ensures process transparency and reduces biases [12,13,14,15,16]. The SHeffield ELicitation Framework (SHELF) is one of the most recognized methods [17,18].

This study represents the first effort to develop a hybrid BN clinical model for predicting survival in patients with pancreatic cancer. This was accomplished by converting expert insights into probability distributions and incorporating established prognostic indicators identified at diagnosis.

## 2. Materials and Methods

### 2.1. Pilot Eliciting Protocol

This study was approved by the local Ethics Committee (109CET). It serves as a pilot experiment in prior elicitation for constructing a BN to model survival outcomes in pancreatic cancer. Prior elicitation, the process of gathering expert opinions to define initial probability distributions, is an essential step for building reliable BNs, especially when clinical data are sparse or lack standardized thresholds. In this pilot phase, we aim to establish a feasible and systematic framework for collecting expert insights that can later be validated and refined in more extensive studies. The results from this pilot will inform the structure and parameters of the BN, setting the basis for future iterations of the model that integrate both expert judgment and empirical data.

This manuscript focuses on developing an expert elicitation protocol to define the prior distributions and structure of a Bayesian network (BN) for predicting pancreatic cancer survival. The BN has not yet been implemented or validated with empirical data. Therefore, no data-driven feature selection or model training has been performed at this stage. Future phases of this research will include BN implementation, validation with real-world datasets, and testing of the model’s predictive capabilities.

Future phases will include scenario testing and expert validation of BN structure to ensure model robustness and clinical relevance further.

### 2.2. Hybrid BN Design

The transparent reporting of a multivariable prediction model for individual prognosis or diagnosis (TRIPOD) checklist [19] is presented in Supplementary Materials S1. The clinical hybrid BN was selected to model the long-term overall survival of pancreatic cancer patients who underwent surgical resection, accommodating both continuous and discretized variables. This decision was based on specific prognostic variables available at the time of diagnosis. The hybrid BN effectively accommodates continuous and categorical variables, preserving important information. The selection of variables for the model was guided by clinical judgment, emphasizing their recognized prognostic relevance for overall survival.

**Figure 1:**
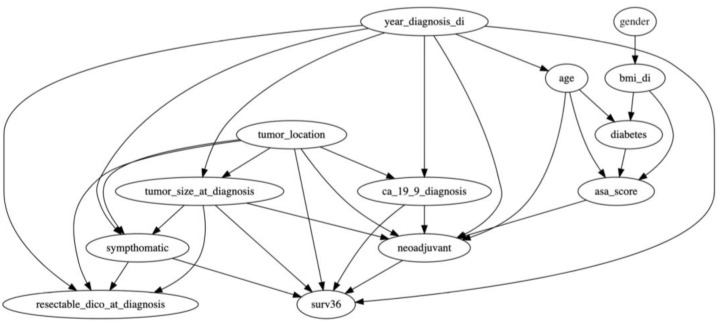
Proposed clinical hybrid Bayesian network.

A single-institution panel of 5 experts in pancreatic cancer care selected well-known and clinically relevant prognostic variables already present at diagnosis. To construct the clinical hybrid BN structure (Figure 1), we specified the Directed Acyclic Graph (DAG) based on clinical relevance and expert insights. The research team initially developed the qualitative structure of the BN, representing conditional dependencies among prognostic variables. This structure was guided by the literature on pancreatic cancer prognosis and refined through iterative consultations with a panel of five experts from the General and Pancreatic Surgery Unit at the Pancreas Institute, University of Verona, a globally recognized center for pancreatic cancer care. These experts have extensive experience and consensus regarding the clinical pathways and prognostic indicators relevant to pancreatic cancer, ensuring that the DAG structure accurately reflects real-world clinical relationships.

Each node in the DAG is a clinically relevant factor, such as CA 19-9 serum levels, tumor size, and resectability status, which are known to impact survival outcomes. The experts validated the DAG’s structure in two phases: first, through individual assessments where each expert reviewed the proposed relationships, and second, in a group session where the qualitative model was discussed and agreed upon. This approach enabled the expert panel to confirm that each connection in the BN represented a realistic clinical dependency, improving the network’s face validity.

No automatic learning methods were used to analyze the available data (for more details, see Supplementary Materials S2). A thorough literature search was conducted to identify suitable variables, focusing on high-quality, peer-reviewed articles published in English.

The following 12 variables, already available at the time of diagnosis, were included in the model:

− CA19.9 serum levels at diagnosis (continuous; expressed as UI/mL);
− Gender (categorical dichotomous; male vs. female);
− Body mass index (BMI; categorical dichotomous; normal/overweight [BMI ≤ 30] vs. obesity [BMI > 30, Kg/m^2^]);
− Year of diagnosis (categorical dichotomous; before 31 December 2014 vs. after 1 January 2015, given the introduction of FOLFIRINOX chemotherapy to clinical practice);
− Tumor location (categorical dichotomous; head vs. body/tail);
− Age (continuous; expressed in years);
− Diabetes (categorical dichotomous; presence vs. absence of diabetes);
− Tumor size (continuous; expressed in millimeters);
− Symptoms (categorical dichotomous; symptomatic vs. no symptoms);
− American Association of Anesthesiology (ASA) Score [20] (categorical dichotomous; ASA I–II vs. ASA III–IV);
− Resectability status (categorical dichotomous; resectable PDAC vs. borderline resectable/locally advanced PDAC according to NCCN criteria, version 2.2021) [21];
− Neoadjuvant treatment (categorical dichotomous; neoadjuvant treatment performed vs. non-performed).

To construct the clinical hybrid BN (Figure 1), the R software [22] (Version 1.3.959, “HydeNet” package) was used. Node-specific model classes were established based on the node class (binary factors, factors with any number of levels, factors with >3 levels, and numeric or integer) [23,24]. The process was manually executed by specifying only the network structure in the call to *HydeNetwork()* or, more specifically, through a list of arguments containing more model objects as elements. In this method, the network structure is automatically built using the names of the response and explanatory variables within each model in the list argument [23,24]. The advantage of this approach is that it increases flexibility in determining the model parametrization for each node. Permissible models include tabulation (*xtabs*), conditional probability table (*cpt*), ordinary least square (*lm*), logistic regression (*glm*), and multinomial logistic regression (*multinom*) [23,24]. After configuring the model’s nodes, the package provides formula-like results of relationships defined in the model.

### 2.3. Elicitation Process—The SHELF Method

To render the BN informative, it was imperative to establish the probability distribution of each node, called the Quantity of Interest (QoI), a priori. When developing the BN, it was essential to establish both marginal and conditional probability distributions for each QoI to represent the dependencies between variables. For each node in the BN, conditional probabilities were elicited given its parent nodes. This approach reflects the BN’s foundational structure, where each variable’s distribution depends on the values of its parent nodes, enabling the model to capture complex interdependencies within clinical variables.

**Figure 2:**
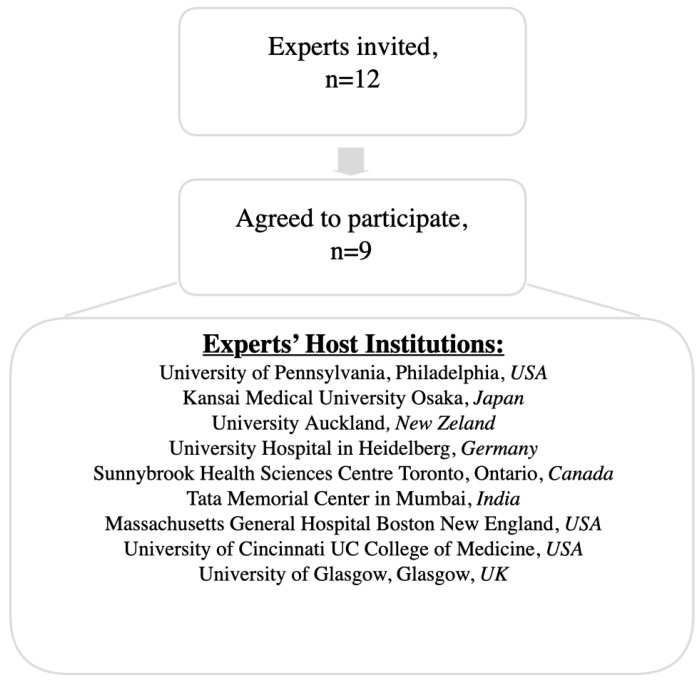
Experts host institutions.

This was achieved through expert judgment to generate a weighted linear pooled distribution using the SHELF method. The following steps were followed according to version 4 of the SHELF workflow: (i) definition of QoIs (where each QoI corresponds to a network variable); (ii) selection of experts (Figure 2 presents the host institutions of the experts involved); (iii) expert training in the QoI elicitation process; and (iv) workshop scheduling.

In addition, we assembled a team that included a coordinator, a facilitator, a recorder, an analyst, and an impartial observer (RIO) [17]. To streamline the elicitation process and refine the survey and QoI, a two-stage protocol consisting of a pilot and definitive phase was implemented, as reported by Morgan [25].

### 2.4. Handling of QoIs

The QoI scores were subjected to an expert-like quartile method [17,26,27,28]. For continuous QoIs, experts were asked to provide credible intervals (ranges), along with the median and lower and upper quartiles. The QoIs were defined based on clinically plausible values. Credible intervals were established to indicate ranges within which the variable’s actual value is expected to lie. However, experts considered it highly unlikely for the true value to fall outside these limits. To reduce overconfidence, these intervals were standardized. For categorical QoIs, experts provided the first, second, and third quartiles of distribution values, representing the “worst” and “best” clinical scenarios, respectively. Following recommendations from the literature, only two of the twelve nodes (year of diagnosis and BMI) were discretized from their original values.

### 2.5. Selection of Experts

**Figure 3:**
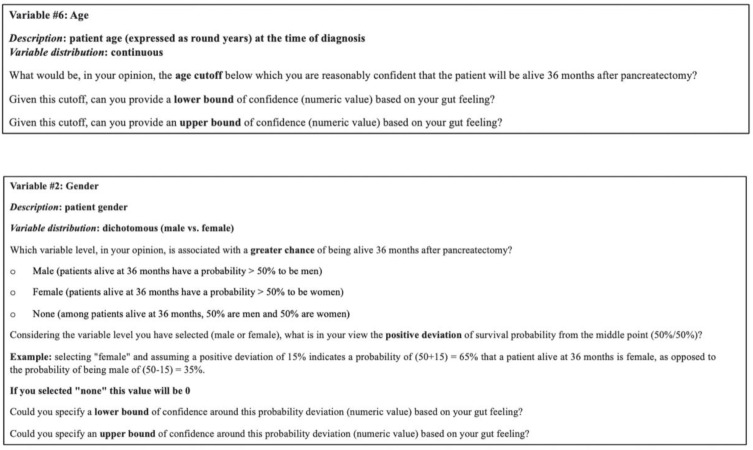
Example of the survey administered to the experts at the first step of the workshop (continuous variable, upper panel; categorical variable, lower panel; See Supplementary Materials S3).

During the pilot phase conducted at the General and Pancreatic Surgery Unit, Pancreas Institute, University of Verona, Verona, Italy, six expert academic surgeons, postdoctoral fellows, and senior residents in surgery were involved, in line with suggestions in the literature [25]. After finalizing the survey and the semantic framework of the QoIs, nine of the initially invited twelve international academic experts in pancreatic cancer care (surgeons) agreed to participate in the project. Once their commitment was confirmed, the experts received an evidence dossier (composed of the initially selected 12 variables, proven by the literature and clinically relevant for prognostic purposes) that summarized contemporary scientific findings related to pancreatic cancer survival prediction and the specific QoIs for which their judgments were sought (the invitation letter can be found in Supplementary Materials S3). The experts then took part in a three-step elicitation process. First, they expressed individual judgments regarding the probability distribution for each QoI. Next, they engaged in a group discussion where anonymized responses were shared. Finally, they collaborated to reach collective decisions on the QoIs, fitting a “consensus” probability distribution to their judgments using SHELF software (https://jeremy-oakley.shinyapps.io/SHELF-multiple/, accessed on 13 January 2025). To enhance user experience, a deidentified web-based survey was employed throughout the process. Expert opinions were solicited to define the prior distributions and dependencies for a BN focused on predicting survival in pancreatic cancer. These questions aim to capture expert judgments regarding the impact of specific variables (e.g., age, gender) on survival probabilities, which are then used to inform the BN structure (see Figure 3; full data available in Supplementary Materials S4).

### 2.6. Pooling Expert Opinions

The judgments were entered into R software, where distributions were fitted to each expert’s input using the best statistical fit method following a complete case analysis as defined in SHELF protocol. The expert opinions were equally weighted, regardless of seniority or confidence, with respect to the node. This approach selected the distribution with the lowest sum of squared deviations between the specified quantiles and those fitted by a least-squares algorithm. The candidate distributions included normal, T, shifted gamma, mirrorgamma, lognormal, log-T, mirrorlog-T, and shifted-scaled beta.

The experts’ elicitation of QoIs was converted into numerical values, which were then interpolated across individual responses and aggregated. Linear mathematical aggregation was used to summarize the data from the expert pool. Visual representations of distribution graphs for each variable were shared with the experts to facilitate consensus on the optimal distributions.

The facilitator then presented the deidentified individual judgments of all experts to the group. The optimal fit distribution was computed as follows: for continuous nodes, the mean and standard deviation of the linear pooled distribution were used. For categorical values, the mean and standard deviation of the pooled distribution were transformed into shape (alpha) and scale (beta) parameters, as specified in Formula (S1) (see Supplementary Materials S5). Additionally, interpolating the beta-pooled distribution was applied to characterize the discrete data provided by experts in quantiles [29,30,31,32,33]. Beta distributions were bound between 0 and 1, aligning with the support of an event rate. Moreover, this distribution offered flexibility to support unimodal, multimodal, and uniform shapes [14]. For continuous nodes, the best-fitting distribution was determined by minimizing the sum of squared residuals.

## 3. Results

**Figure 4:**
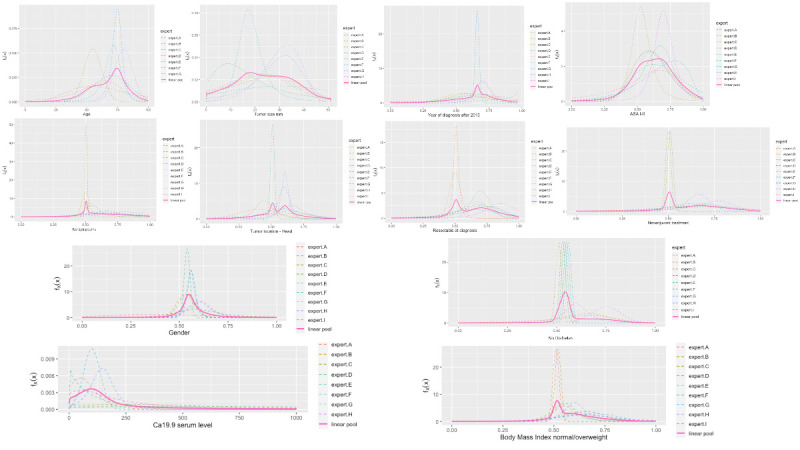
Pooled experts elicited distributions for each of the twelve nodes of the BN.

Figure 4 illustrates the linear pool distribution for each node of the clinical hybrid Bayesian network (BN) developed through the abovementioned process. It presents the elicited probability distributions for each predictor variable, serving as the prior probabilities for each node in the BN. These priors represent the initial beliefs about the possible values of each variable before observing any new patient data based on expert judgments and available demographic or clinical insights. The marginal probabilities are elicited and combined with conditional probabilities in a process explained in Supplementary Materials S6. Each prior distribution in Figure 4 is the starting point for calculating conditional probabilities within the BN. Examples include the following:

- For continuous variables like CA 19-9 serum levels or tumor size, the prior distribution provides a range of plausible values based on clinical experience. This influences the BN’s estimation of survival outcomes for patients with varying CA 19-9 serum levels.
- For categorical variables such as gender, the prior distribution reflects the expected population proportion of male versus female patients, setting an initial baseline for the BN. For example, the probability distribution shown for gender, which lies mostly between 0.5 and 0.6, represents the experts’ pooled estimate of the proportion of male patients among this model’s total population of pancreatic cancer cases. In this case, a value of 0.5 to 0.6 indicates a slight prevalence of one gender over the other, with the probability reflecting the anticipated proportion based on clinical observations or available demographic data.

These prior distributions enable the BN to calculate the conditional survival probabilities by combining the base probabilities (priors) with evidence from related variables. As new data on a specific patient becomes available, the BN updates these probabilities by conditioning on observed values, dynamically refining predictions according to each variable’s influence on survival outcomes (further details in Supplementary Materials S6).

In this way, the priors established in Figure 4 enable the BN to perform probabilistic inference, producing predictions that adjust based on individual patient characteristics. This initial setup of priors is fundamental for model predictions, as it defines how each node relates to others and the likely values each variable may take, ultimately guiding the BN’s predictive accuracy and relevance in clinical settings.

Figure 4 shows a general agreement in distribution shape among the individual experts, although specific disparities were noted in the nodes for tumor size, age, and ASA score.

The informative priors derived from the elicitation process are summarized in Table 1 and Table 2, with details on each node including the pooled expert votes, the best-fit distribution, and the predominant distribution type. The right column indicates the level of expert concordance regarding the distributions of the nodes, represented as the proportion of prevalent distributions to the total distributions. Both tumor size and ASA score exhibit absolute concordance, reflecting the highest level of expert consensus. CA19.9 values and resectability status show a high degree of concordance, while the remaining nodes demonstrate acceptable levels of agreement.

## 4. Discussion

Despite ongoing research efforts, pancreatic cancer continues to have a poor prognosis. In many cases, the disease recurs with a fatal outcome even after surgical resection combined with neoadjuvant or adjuvant treatment [34]. Additionally, pancreatic cancer exhibits significant biological heterogeneity. Some cases display slow and localized growth, while others progress rapidly, highlighting the need for a personalized therapeutic approach [35].

In this context, it is crucial to allocate patients to personalized therapies based on the presumed biology of their disease. This includes decisions such as opting for aggressive surgery followed by chemotherapy versus total neoadjuvant chemotherapy followed by surgical resection. However, existing prognostic tools, such as nomograms, primarily rely on pathological variables—like tumor size and stage [36]—that are often unavailable at the time of diagnosis. Furthermore, these tools typically derive their data from single-center samples, which may limit their generalizability. These models can be classified as traditional or black boxes, both difficult to interpret. Traditional models rely on inferential statistics, drawing information solely from observed data, which makes their inferences highly dependent on the actual sample. However, a deep understanding of the relevant scientific domain suggests that not all scenarios are equally plausible; certain features should be considered a priori more likely than others. This knowledge becomes a valuable resource when available, highlighting the importance of capturing and leveraging it effectively [6]. BNs provide a flexible and unique predictive statistical approach that integrates objective data with subjective opinions, such as expert insights, which can be particularly valuable in a clinical setting. This study represents one of the few attempts to incorporate expert opinions into a prognostic Bayesian framework. The proposed clinical hybrid BN is both innovative and practical, as it enable to integrate existing knowledge regarding continuous and categorical variables within a single network.

In the context of pancreatic cancer management, this integration is especially relevant, as many clinical predictive variables available at diagnosis are continuous (e.g., CA 19-9 serum levels, tumor size from radiology, age), while others are categorical (e.g., gender, tumor location, presence of symptoms, or diabetes). Combining and integrating these heterogeneous variables enables the model to create a comprehensive BN that offers clinicians enhanced tools for accurately modeling long-term survival outcomes. Previous applications of BN in pancreatic cancer did not include expert judgment [8,37]. The SHELF method effectively translates expert opinions into prior probability distributions and accommodates known distribution families. However, when expert consensus on a particular node is suboptimal, parametric methods may need to be more flexible to reflect that node’s nature accurately. In such instances, a semi-parametric approach to prior elicitation should be considered, which offers greater flexibility [38].

The project is in its pilot phase, pertaining to implementing the elicitation process into a BN. Future steps will be developed to make the BN applicable to clinicians for prognostication at diagnosis. From a statistical standpoint, the concordance of the Markov chain algorithm will be tested. Using a real-world dataset including the 12 selected variables, possible clinical scenarios will be created. The sensitivity analysis of these scenarios will be compared and verified by implementing the BN with informative and non-informative priors (a sensitivity analysis plan is presented in Supplementary Materials S6). Further, a user-friendly, interactive application, Shiny, which will assist clinicians in using the developed BN for prognostication purposes, will be developed. Its engagement and ease of use will be measured to assess how well the tool can be integrated into clinical practice, whereas its clinical usability will be measured by assessing how it encourages clinicians to deviate from standard practice when the data suggest a better course of action. Finally, multicenter datasets will be employed to facilitate model validation.

The strengths of this project include its novelty, the intuitive graphical representation of pooled expert distributions, which aids in reaching consensus despite discordance, and the two-phase design that has improved the elicitation process, resulting in clear QoIs and surveys.

However, certain limitations should be acknowledged. First, while the experts were international and not geographically co-located, they all shared backgrounds as surgeons and academics. Despite this commonality, their regular involvement in pancreatic cancer care and research lends reliability to their opinions. Second, the COVID-19 pandemic prevented face-to-face elicitation, as suggested by the SHELF method [26]. Third, to effectively manage the elicitation results, the mean and standard deviation were used for symmetric and continuous variables, while a transformation was applied to derive a beta distribution for categorical variables. These processes may have caused a loss of information. Moreover, this study is an initial pilot phase. Future phases will focus on scenario testing with multicenter datasets and sensitivity analysis, followed by expert-validated scenario testing to ensure robust predictive accuracy for clinical applications.

Finally, some experts did not respond to the invitation, and two nodes did not receive complete responses.

## 5. Conclusions

This project introduces a clinical hybrid BN that integrates expert elicitation, utilizing clinical variables available at diagnosis to model the survival of pancreatic cancer patients. If further validated, this innovative approach can significantly inform clinical decision-making by providing reliably predicted survival outcomes.

## Figures and Tables

**Table 1:** Mean and standard deviation of the linear pool distributions and best fit-derived prior.

Nodes	Mean	SD	Best-Fit Distribution
Ca 19.9 values	130.29	207.84	T (130.29, 207.84, df = 7 ***)
Age (years) *	69.49	15.69	MirrorlogT (69.49, 15.69, df = 6)
Tumor size (mm) **	23.86	11.42	MirrorlogT (23.74, 11.45, df = 7)
Gender	0.56	0.13	Beta (7.62, 5.76)
Body mass index (normal/overweight–obese)	0.59	0.11	Beta (10.07, 6.69)
Year of diagnosis (before 31 December 2014)	0.65	0.65	Beta (3.85, 2.08)
Tumor location (head)	0.54	0.17	Beta (4.26, 3.63)
Diabetes (absence)	0.58	0.11	Beta (12.52, 10.00)
Symptoms (absence)	0.61	0.19	Beta (3.46, 2.15)
American Association of Anesthesiology (ASA) Score I–II	0.62	0.16	Beta (4.75, 2.94)
Resectability at diagnosis	0.64	0.18	Beta (3.83, 2.13)
Neoadjuvant treatment	0.61	0.19	Beta (3.46, 2.22)

* Two experts did not provide a complete response; one did not provide a lower limit and the other only gave null values. ** One expert did not provide the requested values. *** Degrees of freedom are the number of experts minus 1.

**Table 2:** Expert best-fit distribution and type of prevalent distribution of the expert pool.

Nodes	Expert Best-Fit Distribution	Type of Prevalent Distribution (Number of Experts with Prevalent Distribution/Number of Experts)
Ca 19.9 values	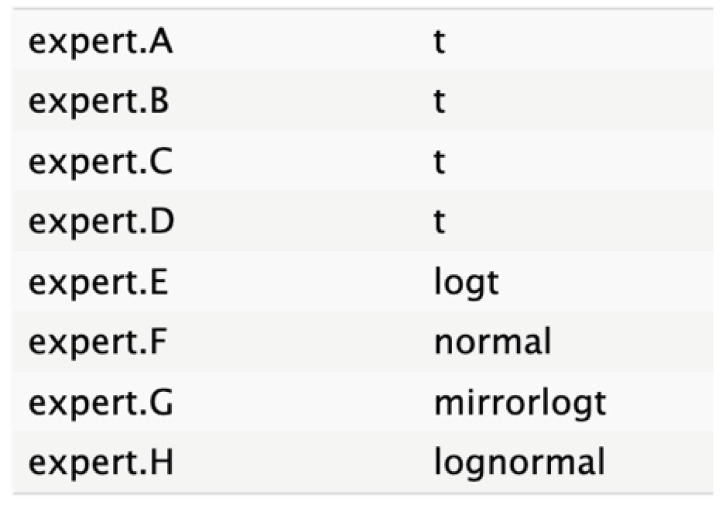	T (6/8)
Age (years)	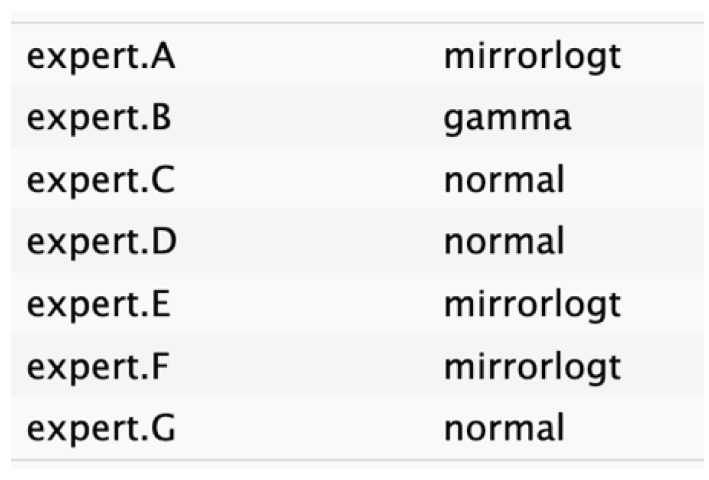	MirrorlogT (4/7)
Tumor size (mm)	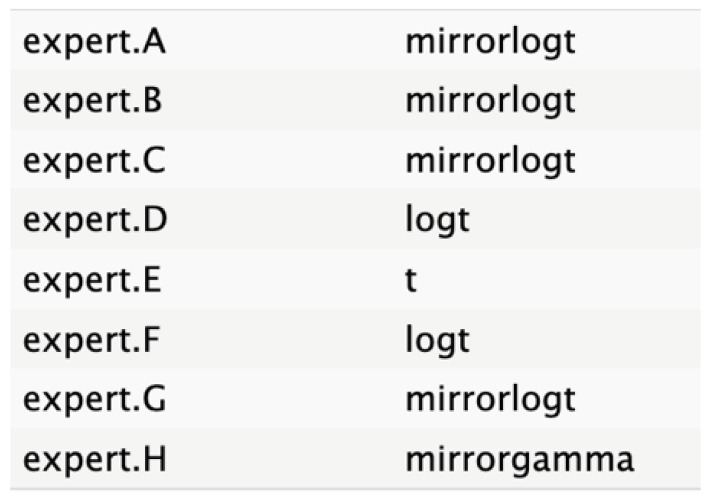	MirrorlogT (8/8)
Gender	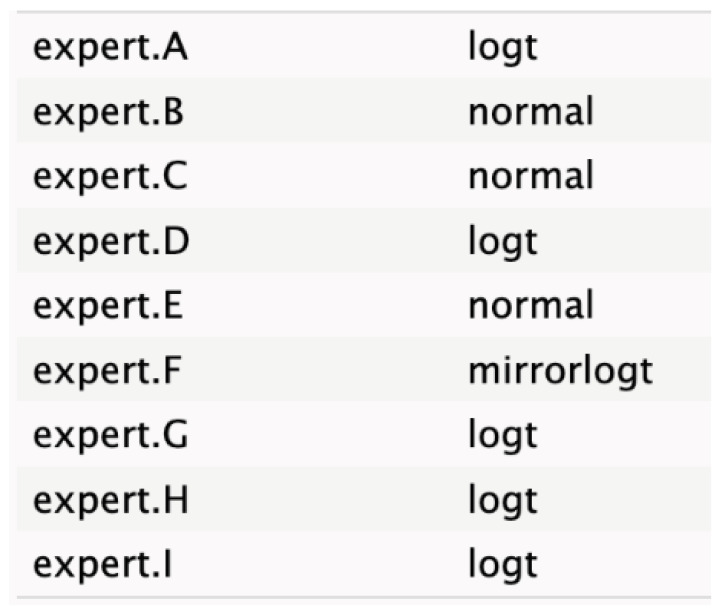	LogT (6/9)
Body mass index (normal/overweight–obese)	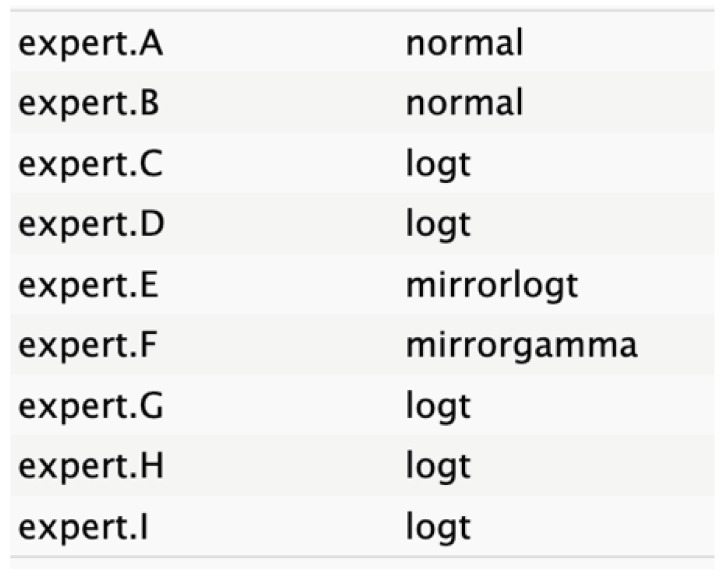	LogT (6/9)
Year of diagnosis (before 31 December 2014)	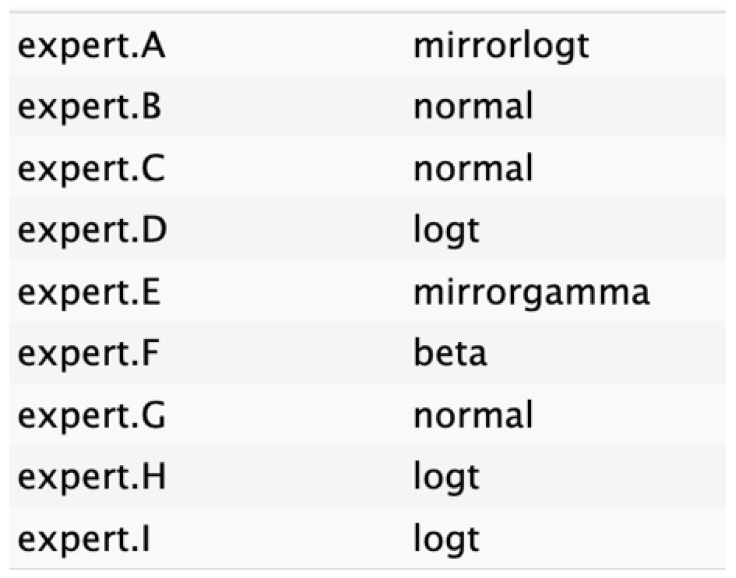	LogT (5/9)
Tumor location (head)	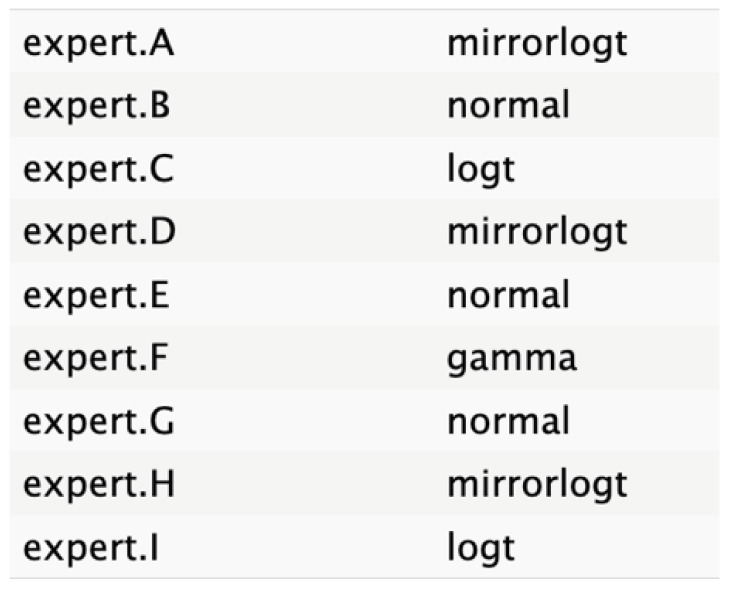	MirrorlogT (6/9)
Diabetes (absence)	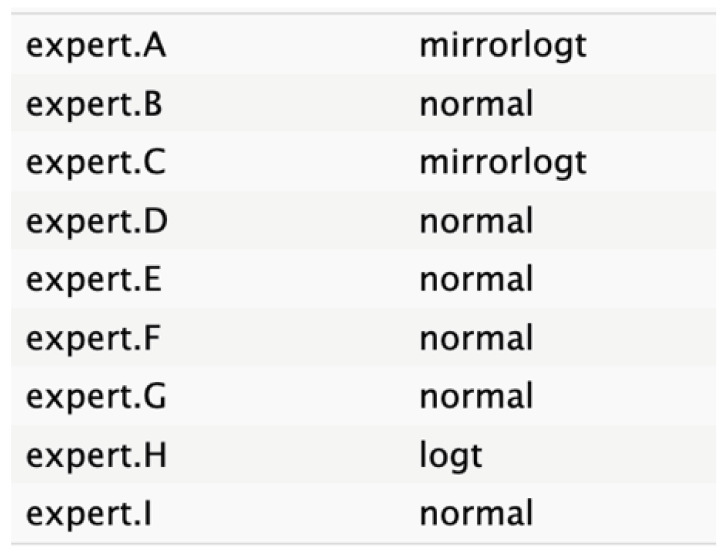	Normal (6/9)
Symptoms (absence)	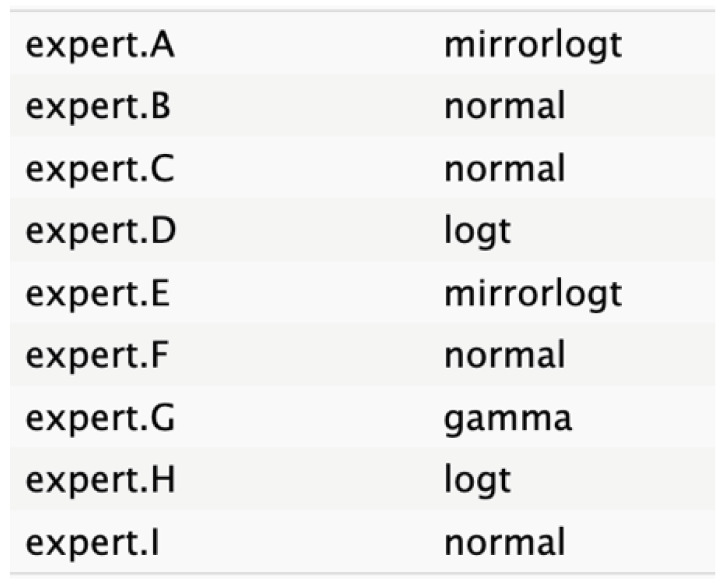	MirrorlogT (5/9)
American Association of Anesthesiology (ASA) Score I–II	** 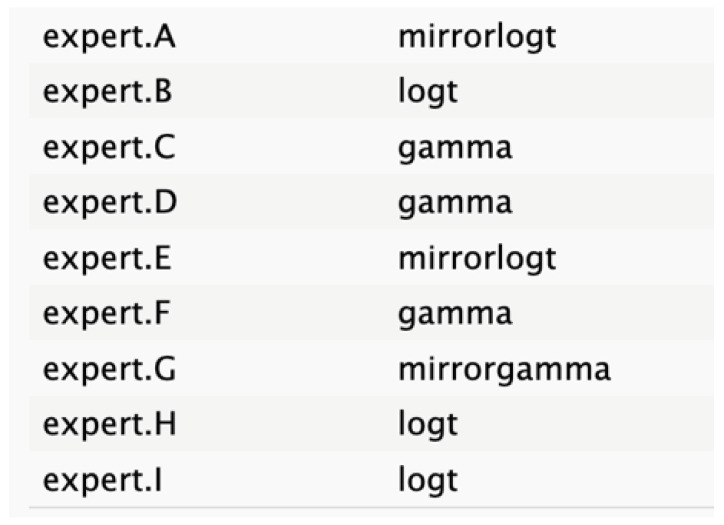 **	LogT (9/9)
Resectability at diagnosis	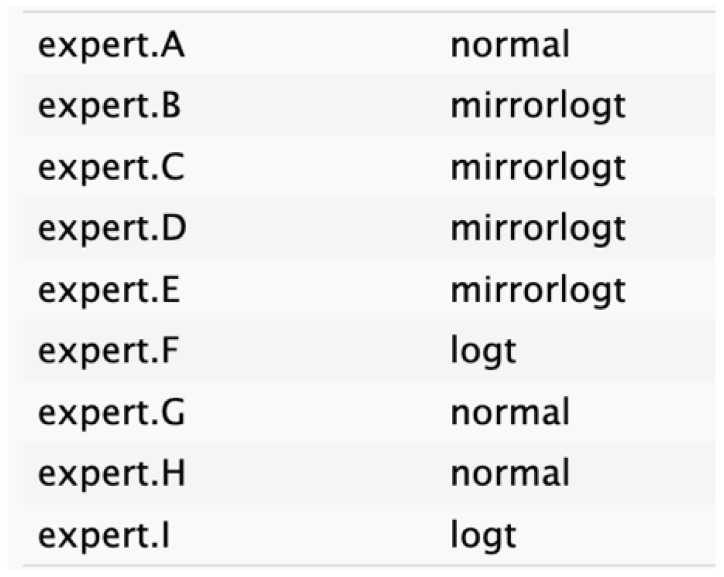	MirrorlogT (7/9)
Neoadjuvant treatment	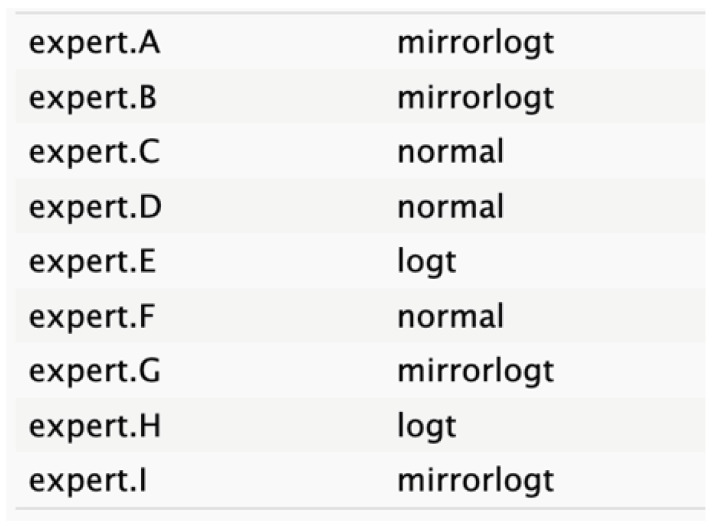	MirrorlogT (6/9)

## Data Availability

The data presented in this study are available in this article and Supplementary Materials.

## Supplementary Materials

The following supporting information can be downloaded at: https://www.mdpi.com/article/10.3390/cancers17020301/s1, Supplementary Materials S1: TRIPOD Checklist: Prediction Model Development; Supplementary Materials S2: Evidence Dossier (Version 1, June 2022); Supplementary Materials S3: Experts invitation letter; Supplementary Materials S4: Survey administered to experts at first step of workshop; Supplementary Materials S5: Formula (S1); Supplementary Materials S6: Technical Appendix.

## Abbreviations

BN, Bayesian network; QoI: Quantity of Interest.
