# Supplementary material for: Expert Judgment Supporting a Bayesian Network to Model the Survival of Pancreatic Cancer Patients
Source: Cancers (Basel). 2025 Jan 17;17(2):301. doi: 10.3390/cancers17020301 (PMC11764457; doi:10.3390/cancers17020301)
Supplement: Supplementary file 1 [file cancers-17-00301-s001.zip › Supplementary Materials S2.pdf]

Supplementary Materials S2

*Evidence Dossier (*Version 1, June 2022)

Wider context

Prognosis of pancreatic cancer is dismal. Despite being considered the only chance of cure, surgical resection of primary tumor mostly fails achieving complete tumor clearance. In fact, both local and systemic disease recurrence are common following surgery, with 80% of patients relapsing within 2 years and showing a median 7.5 month survival thereafter.[1] Multiple survival prediction models have been developed thus far to forecast disease free and overall survival, encompassing clinical and laboratory parameters along with pathological features. However, such nomograms have demonstrated modest performance overall[2,3] and no applicability into daily practice.

Whether unique patient- and tumor-specific characteristics evident at the time of diagnosis effectively impact survival after surgery - and how they interplay - is still largely unknow. Decoding causal relationships between baseline parameters and survival could help decision making and treatment selection.

In order to build a clinical Bayesian Network model capable to unravel causal relationship between baseline parameters and survival, a priori probability distribution of such variables has to be determined according to clinical evidence. Therefore, we are interested in the following question: <u>in your opinion</u>, <u>what the chance is that a given value of certain informative parameters (*Quantities of Interest*, *QoIs*) manifest at the time of PDAC diagnosis predicts the likelihood of being alive 36 months after surgery?</u>

<u>Quantities of Interest (*QoIs*)</u>

Ca19.9

Extended definition: *Ca19.9 serum level (expressed as UI/mL) measured at time of first PDAC diagnosis (i.e. first Ca19.9 measurement available after diagnosis of PDAC was achieved/strongly suspected).*

Variable distribution: continuous.

Question(s): in your opinion, which is median baseline Ca19.9 probability distribution for patients alive 36 months after surgery? Which the upper and lower quartile thresholds are for the same patients?

Ca19.9 is the primary, validated biomarker of PDAC, with some 80% of patients with advanced disease showing an increase of such antigen (>37 U/mL). Quantitative assessments are employed to assess disease progression, burden and response to treatment. Beyond PDAC, Ca19.9 levels typically rise in the presence of biliary obstruction and cholangitis, pancreatitis or other gastrointestinal and hepatobiliary tumors, thus showing poor specificity. Moreover, up to 10% of Caucasian population does not produce such an antigen because of the deficiency of fucosyl-transferase enzyme (which is also responsible in the secretion of red blood cells Lewis Antigen A), and they show persistently undetectable or below 1 UI/mL measurements.

Preoperatively, Ca19.9 correlates with resectability status and AJCC staging, although there is no formal consensus yet regarding baseline thresholds to inform decision making. According the results of a seminal paper by Hartwig et al.[4] linking preoperative Ca19.9 levels to resection rate and survival in 1543 patients, both the 2015 Society of Medical Oncology (ESMO) Clinical practice guidelines[5] and the 2017 International Association of Pancreatology consensus on borderline resectable (BR) PDAC[6] set the 500 U/mL threshold as indicative of suspected occult metastatic dissemination and poorer prognosis.

However, even lower baseline values (i.e. 250 and 100 U/mL) have been associated with the presence of metastatic disease during staging laparoscopy.[7,8] Similarly, more recent reports indicate that patients having Ca19.9 ≥80 and 100 UI/mL at diagnosis showed significantly reduced survival following neoadjuvant chemotherapy plus surgery for both (borderline) resectable[9] and locally advanced[10] PDAC, compared to those with Ca19.9 below such thresholds.

Gender

Extended definition: *patient gender expressed as male vs. female.*

Variable distribution: dichotomous (*male vs. female)*.

Question(s): in your opinion, which the likelihood (expressed as %) of being male rather than female for patients alive 36 months after surgery?

Which the best and the worse scenario are for the same patients?

Incidence of PDAC is reported to be generally higher in men than in women worldwide (age standardized rates: 5.7 vs. 4.1/100.000, respectively).[11] However, no significative difference in terms of mortality emerges with respect to gender according to the latest WHO Globocan initiative and Cancer Statistics reports.[11,12] A recent study by Pijnappel [13] et al. including some 7500 Dutch patients with metastatic pancreatic cancer found that female were more likely to have longer overall survival (HR 0.89, 95%CI 0.84-0.93) after adjusting for confounders. As a matter of fact, implications of patient gender on pathogenesis, treatment response and prognosis of PDAC are still largely unknown.

Body Mass Index

Extended definition: *Body Mass Index (BMI) calculated as patient weight measured at diagnosis (kilograms) divided by square height (meters).*

Variable distribution: dichotomous (normal/overweight [BMI ≤30] vs. obesity [BMI >30]).

Question(s): in your opinion, which the likelihood (expressed as %) of being normal weight (BMI ≤30) rather than obese (BMI >30) at diagnosis for PDAC patients alive 36 months after surgery? Note: sum of expressed likelihoods must equal 100%.

Which the best and the worse scenario are for the same patients?

Obesity is a universally recognized risk factor for PDAC (+35% estimated incidence of PDAC in obese individuals). Multiple biological pathways enhanced by visceral adipose tissue are deemed to be involved in the pathogenesis of pancreatic cancer, such as inflammation, oxidative stress and cell proliferation.[14,15] In 2001 Michaud et el.[16] reported a risk-adjusted 1.72 Relative Risk (95% CI 1.19-2.4) of developing pancreatic cancer for obese patients compared to those with BMI <23%, whereas other Authors postulated that a 10cm increase in waist circumference are enhances PDAC risk of 11%.[14]

A linear association has been recognized between escalating BMI and worse prognosis, with overweight and obese adults showing significantly reduced survival (HR 1.26 [95% CI 0.94-1.69], p=0.04; and HR 1.86 [95%CI 1.35-2.56], p<0.001, respectively) after adjusting for several confounders.[17] Besides promoting pancreas carcinogenesis, obesity can impair prognosis of PDAC patients undergone pancreatic resection throughout indirect mechanisms. For instance, obesity strongly correlates with increased morbidity following pancreatoduodenectomy.[18] If on the one hand obese patients are more prone to develop cardiac, respiratory and infective complications overall, visceral adiposity entails soft pancreatic texture, and, in turns, pancreatic fistula development,[19,20] which has been demonstrated as independent predictor of worse survival after neoadjuvant treatment for PDAC (overall survival: 17 vs. 34 months, p=0.002; Hazard Ratio 2.80 95%CI 1.44-5.45, p=0.002).[21]

At the other end of the spectrum, pancreatic cancer patients often show malnutrition, cachexia and sarcopenia, which are proven risk factors for chemotherapy-induced toxicities as well as postoperative complications and attrition.[22] Persistent weight (HR 1.55) and muscle mass (HR 1.49) loss are negatively associated with survival in patients receiving induction treatment for locally advance disease.[23] However, while being ubiquitously employed, BMI calculation has repeatedly proven poor performance in nutritional derangement detection[24] and prognosis evaluation,[25] since a low or underweight BMI do not usually equate to a worse overall survival for pancreatic cancer.

Year of Diagnosis

Extended definition: *Year of first diagnosis of PDAC*.

Variable distribution: dichotomous (before December 31st 2014 vs. after January 1st 2015).

Such time threshold is set given the implementation of multiagent chemotherapy regimens in the clinical practice. Since Gemcitabine-NabPaclitaxel was approved for the treatment of metastatic PDAC in 2013, it could be presumed that such regimens were significantly employed from 2015 onwards.

Question(s): in your opinion, which the likelihood (expressed as %) of being diagnosed before December 31st 2014 rather than after January 1st 2015 for PDAC patients alive 36 months after surgery? Note: sum of expressed likelihoods must equal 100%.

Which the best and the worse scenario are for the same patients?

Pancreatic cancer prognosis has improved in recent years overall, with all-stage 5-year survival rate increasing from 6% between 2001-2007[26] to 11% between 2011-2017.[27] In contrast to the marginal improvements observed for patients with metastatic disease, significant advancement have been obtained for those with localized and regional disease, who are currently expecting a 42 and 14% 5-year survival rate, respectively.[27]

Such achievements are primarily due to the implementation of multimodal peri-operative treatments in the last decade, which have significantly extended indications for surgery and, in turn, the chance to obtain disease control. The most significant results have been gained with the introduction of multi-agent chemotherapy regimens FOLFIRINOX (2011)[28] and Gemcitabine plus Nab-Paclitaxel (2013)[29], initially employed in the metastatic setting. Notably, a 23% 3-year survival rate was observed for patients with locally-advanced/metastatic PDAC at diagnosis receiving neoadjuvant treatment (30% FOLFIRINOX) plus surgical resection,[30] thus opening the door to the use of such regimens to accomplish downstaging before surgical exploration.

With respect to adjuvant treatment, Gemcitabine monotherapy had represented the standard of care since its approval in late 1990s, providing a 20.7% 5-year survival rate according to the CONKO-001 randomized trial.[31] Starting from 2018, FOLFIRINOX has been introduced as first-choice adjuvant chemotherapy regimen, in the light of a median 54.4 month overall survival (63.4% 3-year survival rate) obtained in the seminal PRODIGE24 trial by Conroy et al.[32] Of note, the sharp prognosis improvement observed in the Gemcitabine-alone arm (median OS: 35 months; 3-year survival rate: 48.6%) compared to historical data sheds light on broader refinement in pancreatic cancer care achieved in recent years – encompassing, among the others, symptom palliation, surgical technique and treatment of recurrence.

Tumor Location

Extended definition: *Primary tumor location within the pancreatic gland, expressed as 'head' (also including uncinate process and isthmus neoplasms) vs.'body-tail', as indicated during preoperatory assessment.*

Variable distribution: dichotomous (head vs. body-tail).

Question(s): in your opinion, which the likelihood (expressed as %) of harboring cancer in the pancreatic head rather than in organ body or tail, for patients alive 36 months after surgery? Note: sum of expressed likelihoods must equal 100%.

Which the best and the worse scenario are for the same patients?

The degree to which tumor location affects pathologic characteristics and prognosis of PDAC after surgical resection has not been established yet. Substantial differences between head and body-tail cancers may rise when considering time to disease presentation and diagnosis, vascular contact/infiltration, invasion of surrounding organs, spread to lymphatic basins, as well as complication burden following surgical resection.
Contrasting conclusions regarding the prognosis of resected head and body-tail cancers have been published thus far. A recent analysis[33] including some 1460 patients from two high-volume institutions found no significant differences between cephalic and distal cancers in terms of both overall and disease free survival after adjusting for confounders, although body-tail cancers displayed bigger size and significantly lower rates of G2/G3 grading and perineural invasion. Dreyer et al.[34] reported a significantly worse prognosis for patients with body-tail neoplasms from the Australian Pancreatic Cancer Genomic Initiative (n=518 patients; 12.1 vs. 22 months, p=0.001; HR 1.72 95%CI 1.31-2-26; p<0.001), mainly due to the higher incidence of the squamous subtype in such a cohort compared to cephalic neoplasms. Conversely, when analyzing the National Cancer Database (n= 40.980 patients), Winer et al.[35] found a significant survival reduction for patients with head PDAC compared to those with body (HR 0.92, 95%CI 0.87-0.98, p=0.006) and tail tumors (HR 0.83, 95%CI 0.79-0.87, p<0.001).
With such inconsistencies in mind, whether some biological differences actually exist between tumors harboring from pancreatic head and body-tail, needs further scrutiny.

Age

Extended definition: *Patient age (expressed as round years) at the time of primary tumor diagnosis.*

Variable distribution: Continuous

Question(s): in your opinion, which is the median age probability distribution for patients alive 36 months after surgery?

Which the upper and lower quartile thresholds are for the same patients?

With median age at diagnosis being 71 years old, little is known regarding the impact of age on prognosis of resected PDAC patients. To date, there is no strong evidence indicating differences in tumor biology according to age at presentation, with the exception of some pathogenic germline mutations associated with earlier onset (i.e. BRCA1/2 and MMRs variants) and improved survival[36]. However, such conclusions have not been confirmed by other studies.[37,38]

Historical surgical data from Johns Hopkins Hospital (1975-2009) suggest slightly improved survival for patients <45 years old undergoing resection compared to those ≥70 years old (n=945 patients; median Overall Survival 19.0 vs. 16.0 months, with actual 5-year survival rate 24 vs 11% [p<0.05], respectively).[39] Similarly, Ordonez et al.[40] found that patients <50 years old within the 2004-2013 National Cancer Database had a marginal OS improvement as compared to older counterparts (27.3 vs. 24.3 months; late-onset PDAC: HR 1.076, p<0.001), while an Australian population-based study[41] concluded that patients 70-80 years old suffered poorer prognosis compared to the ≤60 years old cohort (HR 1.33, 95%CI 1.04-1.69).

Other studies have observed comparable survival across age groups following pancreatic resection for PDAC, as concluded by Van der Geest et al.[42] investigating the 2005-2013 Netherlands Cancer Registry (n=3845 patients). Such results are in line with the conclusion of a recent study analyzing the SEER database,[43] demonstrating how all-cause death after resection steadily increases with age at diagnosis, while cancer-specific death rates tended to decrease across age groups at a much slower pace. Therefore, it might be inferred that there is a limited contribution of cancer-related mortality to the observed survival trends, when compared to the risk of death of other causes.

Diabetes

Extended definition: *Presence of diabetes (any casual blood sugar ≥200 mg/dL / 11.11 mmol/L) at the time of diagnosis.*

Variable distribution: Dichotomous.

Question(s): in your opinion, which the likelihood (expressed as %) of suffering of diabetes at the time of diagnosis (regardless time of onset), for patients alive 36 months after surgery?

Which the best and the worse scenario are for the same patients?

Diabetes mellitus is a universally recognized risk factor for pancreatic cancer development. However, contribution of diabetes on PDAC prognosis remains unclear. A recent study from the Massachusetts General Hospital reported that overall survival following PDAC surgery almost halved compared to non-diabetic patients (18 vs. 34 months; p<0.001).[44] A recent meta-analysis[45] of 17 studies encompassing 5407 patients undergone pancreatic resection (among which 1669 with a diagnosis of diabetes at diagnosis) concluded that diabetes significantly contributed to worsen overall survival on multivariable analysis (RR 1.24, 95%CI 1.05-1.45, p=0.01). Interestingly, a stratification analysis by diabetes subtypes revealed that new-onset diabetes (i.e. ≤2 years before PDAC diagnosis), but not the long-standing variant, was associated with reduced survival. Moreover, diabetic patients did not show increased overall morbidity, mortality nor risk of pancreatic fistula development, but rather had larger tumor size. A previously published meta-analysis focusing on patients with resectable pancreatic tumors also displayed worse overall survival following complete resection (HR 1.32, 95%CI 1.46-1.60).[46] Moreover, Hartwig et al.[47] reported an association between insulin-dependent diabetes and poorer prognosis after surgery for PDAC (HR 1.28 95%CI 1.11-1.72, p=0.009), whereas no significant survival difference was observed between non insulin-dependent diabetic and non-diabetic individuals.

Grounding on these premises, multiple studies have investigated the implementation of anti-diabetic drugs to improve survival of PDAC patients. Despite initial promising results observed in the nonmetastatic setting, no survival benefit for metformin administration in diabetic patients was observed after adjusting for the duration of treatment following cancer diagnosis.[48]

Tumor size

Extended definition: *Greater axial dimension of primary tumor (expressed in millimeters) as measured at imaging at the time of diagnosis. In the case of multiple measurements available, the greatest size measurement is considered.*

Variable distribution: Continuous.

Question(s): in your opinion, which is the median tumor size probability distribution for patients alive 36 months after surgery?

Which the upper and lower quartile thresholds are for the same patients?

As is the case of most solid neoplasms, pathological tumor dimension segregates with prognosis of pancreatic cancer. However, evidence is scant regarding the prognostic impact of tumor size assessed through cross-sectional imaging at the time of diagnosis, nor such a parameter has been incorporated in the definition of resectability. It has been calculated that imaging could underestimate tumor size by around 20% when compared to gross pathology.[49] Moreover, in the same study comparing the contribution of tumor size when measured by either imaging or pathology on overall survival, only the latter was negatively associated with prognosis (HR 1.26, 95%CI 1.03-1.5, p=0.02), with tumors ≥2cm being also associated with higher Ca19.9 levels, lymph node metastasis and incomplete resection.[49]

Notably, it should be kept in mind that PDAC of the body and tail usually present with greater dimensions compared to those arising in the head – likely because of a delay in symptoms development. A recent study from Japan[50] highlighted that in 158 resected PDAC of body and tail with no evident vascular abutment/infiltration, tumors ≥4cm had higher rates of extra-pancreatic infiltration (19 vs. 2%), incomplete resection (16 vs. 4%), as well as higher risk for early disease recurrence (HR 6.5, 95%CI 2.34-18.1, p<0.001) and poorer survival (HR 2.07, 95%CI 1.30-3.29, p=0.001) on multivariable analysis. Therefore, the authors suggest abandoning upfront surgery in the case of anatomically resectable distal PDAC larger than 4cm, but rather seeking for downsizing through neoadjuvant approaches. Of note, comparable results were reported by Li et al.[51], who identified the 4cm tumor size cutoff measured through preoperative contrast-enhanced CT scan as predictor for both disease free and overall survival (HR 2.70 95%CI 1.32-5.52, p=0.006) when considering together head and body-tail cancer.

Symptoms

Extended definition: *Presence of any of the following at the time of diagnosis: jaundice, significative weight loss (≥10% in the last six months), gastric outlet obstruction, typical epigastric pain, low back pain.*

Variable distribution: Dichotomous.

Question(s): in your opinion, which the likelihood (expressed as %) of complaining any of the abovementioned symptoms at diagnosis, for patients alive 36 months after surgery?

Which the best and the worse scenario are for the same patients?

Because symptoms of PDAC are vague and unspecific, there frequently occurs a delay in seeking medical attention, with the result that diagnosis of PDAC is mostly made only at advanced stages. At the time of consultation with the surgeon/medical oncologist, patients often suffer from intense abdominal or back pain, significant weight loss due to anorexia and gastric outlet obstruction, or jaundice, showing impaired performance status and disability. Therefore, the presence and intensity of symptoms could effectively impact on prognosis, influencing the capability to receive multiagent chemotherapy regimens and surgery.

However, few studies have investigated the contribution of symptoms on survival, with contrasting results. A large study involving some 1500 patients undergone resection for PDAC detected only a marginal impact of symptoms on overall survival (HR 1.32, 95%CI 1.005-1.749, p=0.046), which was not confirmed on multivariable analysis. Similarly, no effect of clinical presentation (i.e. jaundice, weight loss and abdominal pain, considered both together and in isolation) was observed in another study collecting patients operated upon between 1997 and 2002.[52]

Conversely, an association between back pain and worse prognosis following surgery was first described by Kelsen et al.[53] and lately confirmed by Brennan et al.[3] (HR not available), with the presence of such a symptom being incorporated into a predictive model along with multiple pathological variables. Moreover, a recent study by Tekikawa et al.[54] confirms that PDAC patients who are asymptomatic at diagnosis are more likely to have smaller tumor size, anatomically resectable tumors, earlier disease stage, as well as improved survival after surgery (3-year survival rate: 41.7 vs. 15.3%, p<0.001), as suggested by previous smaller reports.[55,56]

American Association of Anesthesiology (ASA) Score

Extended definition: *ASA score (grouped as ASA I-II and ASA III-IV) attributed at the time of diagnosis.*

Variable distribution: Dichotomous.

Question(s): in your opinion, which the likelihood (expressed as %) of having a ASA score III-IV at diagnosis, for patients alive 36 months after surgery?

Which the best and the worse scenario are for the same patients?

ASA classification of physiological status was developed for practical categorization of operative risk in surgical patients. Moreover, ASA classes have been demonstrated to segregate with unique survival cohorts for several tumors. Despite being a universally utilized tool, association between baseline ASA score and PDAC survival has not been extensively investigated, and results remain conflicting.

Interestingly, ASA score is the only clinical, non-biological element of the prognostic HELPP-score recently developed by the Heidelberg group.[57] In fact, after analyzing a large cohort of patients (n=1197) with (borderline) resectable PDAC candidate for surgery, ASA class was significantly associated with survival, with ASA III-IV patients showing lower 3-year survival rate compared to those with a ASA class I-II (29.5 vs. 19.3; p<0.001). On the other hand, ASA classes displayed no correlation with survival in another contemporary series of some 1500 patients undergone pancreatoduodenectomy for PDAC.[58]

Resectability Status

Extended definition: *Anatomical resectability status defined according to the National Comprehensive Cancer Network (NCCN) Practical Guidelines for Pancreatic Adenocarcinoma criteria at the time of diagnosis.*

Variable distribution: Dichotomous (resectable PDAC vs. borderline resectable/locally advanced PDAC according to NCCN criteria)

Question(s): in your opinion, which the likelihood (expressed as %) of having a radiologically resectable disease at diagnosis rather than borderline resectable/locally advanced at diagnosis, for patients alive 36 months after surgery? Note: sum of expressed likelihoods must equal 100%.

Which the best and the worse scenario are for the same patients?

With the aim of solving ambiguities of daily practice terminology, criteria for the definition of resectability status at diagnosis have been at first proposed by a AHPBA/SSAT/SSO/SCCG consensus (2008)[59], and subsequently included into NCCN guidelines (2015).[60]

The notion of 'anatomical' resectability, based on the degree and extent of peri-pancreatic vessel infiltration observed at preoperative imaging, is intended to express the likelihood of obtaining margin free tumor resection – a prerequisite for cure of pancreatic cancer. Therefore, allocation into NCCN resectability classes (i.e. resectable, borderline resectable and locally advanced-unresectable disease) implies, at least theoretically, gross differences in prognosis.

Current data endorsing this dogma are ambiguous. To date, patients with resectable disease might achieve astounding survival goals according to the results of the PRODIGE-24 trial[32] (median OS: 54.4 months). Whether such results are realistic also for borderline resectable patients is yet to be demonstrated. However, among patients enrolled into the PREOPANC-1 trial, no significant OS differences emerge between resectable and BR patients receiving preoperative chemo-radiation[61], although that was not an endpoint of the study.

Conversely, LA pancreatic cancer has traditionally been considered an incurable disease, with a 5-year survival rate about 7-12%.[62,63] However, for highly selected patients who achieve resection (<10-20%), contemporary real-world data indicate a 47.3 month median Disease Free Survival - even longer compared to BR patients receiving neoadjuvant treatment plus resection (median DFS: 35.4 months).[63] A recent study from the Karolinska Institute[64] confirms such results, showing no substantial variance of survival between initially BR and LA patients (median OS: 31.9 vs 21.8 months; 3-year survival rate: 43.6 vs. 39.5%, respectively). Of note, some 80% of patients received venous/arterial resections in this series. Similarly, resectability status at diagnosis was not a predictor of survival among resected BR/LA patients in another study by Reni et al. (median OS: 29.1 vs. 30.0 months; 2-year survival rate: 64.4% vs. 61.5%, respectively).[65]

Such data would suggest that extent of peripancreatic vessel infiltration, while determining the chance to undergo surgery, has modest biological meaning once tumor resection is achieved. Therefore, the notion that BR and LA cancers constitute distinct prognostic groups *per se* might be debunked.

Finally, given some biases in data collection and analysis, some caveat is necessary when comparing results of such studies. First, heterogeneity of survival outcomes might be attributable to multiple competing causes besides biological differences between groups (so called *selection* and *allocation biases).* In fact, variation in the indications for surgery, technical skills and audacity of the surgeon, as well as diversity of peri-operative treatments employed can act as significant confounders. Moreover, most studies merge BR patients within either resectable or LA cohorts alternatively, thus impairing generalizability of conclusions. In addition, subjectivity in defining resectability status on imaging must be accounted for (*misclassification bias*). As a matter of fact, notable disagreement often exists between observers,[66] and limitations in predicting actual resectability from imaging review are compelling also for life-long expert pancreatic surgeons – as enlightened by a thought-provoking study by Ferrone et al.[67]

Neoadjuvant treatment

Extended definition: *Completion of at least 3 months of preoperative chemotherapy, with or without radiation therapy.*

Variable distribution: Dichotomous.

Question(s): in your opinion, which the likelihood (expressed as %) of having received preoperative neoadjuvant treatment for patients alive 36 months after surgery? Note: sum of expressed likelihoods must equal 100%.

Which the best and the worse scenario are for the same patients?

Advantages of preoperative chemo(radiation)therapy for BR and LA patients are documented by countless reports nowadays. Cytoreductive therapy plus surgery represents the only chance to lengthen survival for LA patients (see above).

In the (borderline) resectable setting neoadjuvant approach is deemed to provide significant survival improvement,[68] with upfront resection for BR disease being no longer recommended as of the 2016 NCCN Practice Guidelines. The PREOPANC-1 RCT[61] evaluated neoadjuvant Gemcitabine with sequential radiation therapy vs. immediate surgery in 246 patients with resectable (54%) or BR (46%) PDAC. Despite no significant survival differences in the initial intention-to-treat analysis, subgroup analyses showed some benefits of preoperative treatment for patients with initial BR disease (median OS: 17.6 vs. 13.2 months; R0 rate: 79 vs. 13%), and for those who started adjuvant chemotherapy (median OS: 35.2 vs. 19.8 months). Furthermore, recently published long-term results of the trial also reveal significantly higher 3-year survival rates for the neoadjuvant arm vs. upfront surgery (27.7 vs. 16.5%),[69] which are confirmed when also considering resectable and BR cohorts separately.

In line with current standard-of-care treatment protocols for PDAC, multiagent treatments are now being tested in the neoadjuvant setting. For instance, the PREOPANC-2 trial[70] is recruiting resectable and BR patients to test the efficacy of peri-operative Gemcitabine with radiation therapy versus total neoadjuvant FOLFIRINOX, while neoadjuvant mFOLFIRINOX with or without radiation is under scrutiny for BR patients in the Alliance A021501 trial.

Important controversies remain regarding the utility of neoadjuvant treatment for initially resectable disease, especially when compared to the optimistic results provided by upfront surgery plus adjuvant FOLFIRINOX in the PRODIGE-24 trial.[32] The small PACT-15 trial[71] has suggested the benefit of peri-operative PEXG regimen versus adjuvant PEXG or Gemcitabine (3-year survival rate: 55 vs. 43 vs. 35%, respectively). Conversely, the SWOG S1505 trial[72] failed to prove superiority of both preoperative mFOLFIRINOX and Gemcitabile-NabPaclitaxel (estimated 2-year survival rate: 47 and 23.6%, repectively) compared to historical data from adjuvant trials (null hypothesis of estimated 2-year survival rate: 40%). A recent sequential

analysis of such RCTs (along with other trials prematurely stopped) reveals no superiority of neoadjuvant strategy versus upfront surgery (RR for mortality per 100 person-years: 0.83, 95%CI 0.64-1.08; p=0.167), with only 18% of the sample size needed to demonstrate survival improvement being reached thus far. Multiple RCTs are currently ongoing to establish the efficacy of neoadjuvant approaches for resectable PDAC (NorPACT- 1, PANACHE01, PREOPANC-3, Alliance A021806…).

References

1. Groot VP, Gemenetzis G, Blair AB, et al. Defining and Predicting Early Recurrence in 957 Patients With Resected Pancreatic Ductal Adenocarcinoma. *Ann Surg.* 2019;269(6):1154-1162.

2. Huang L, Balavarca Y, van der Geest L, et al. Development and validation of a prognostic model to predict the prognosis of patients who underwent chemotherapy and resection of pancreatic adenocarcinoma: a large international population-based cohort study. *BMC Med.* 2019;17(1):66.

3. Brennan MF, Kattan MW, Klimstra D, Conlon K. Prognostic nomogram for patients undergoing resection for adenocarcinoma of the pancreas. *Ann Surg.* 2004;240(2):293-298.

4. Hartwig W, Strobel O, Hinz U, et al. CA19-9 in potentially resectable pancreatic cancer: perspective to adjust surgical and perioperative therapy. *Ann Surg Oncol.* 2013;20(7):2188-2196.

5. Ducreux M, Cuhna AS, Caramella C, et al. Cancer of the pancreas: ESMO Clinical Practice Guidelines for diagnosis, treatment and follow-up. *Ann Oncol.* 2015;26 Suppl 5:v56-68.

6. Isaji S, Mizuno S, Windsor JA, et al. International consensus on definition and criteria of borderline resectable pancreatic ductal adenocarcinoma 2017. *Pancreatology.* 2018;18(1):2-11.

7. Alexakis N, Gomatos IP, Sbarounis S, et al. High serum CA 19-9 but not tumor size should select patients for staging laparoscopy in radiological resectable pancreas head and peri-ampullary cancer. *Eur J Surg Oncol.* 2015;41(2):265-269.

8. Karachristos A, Scarmeas N, Hoffman JP. CA 19-9 levels predict results of staging laparoscopy in pancreatic cancer. *J Gastrointest Surg.* 2005;9(9):1286-1292.

9. Tsai SS, George B, Wittmann D, et al. Importance of Normalization of CA19-9 Levels Following Neoadjuvant Therapy in Patients With Localized Pancreatic Cancer. *Annals of Surgery.* 2020;271(4):740-747.

10. Michelakos T, Pergolini I, Castillo CF, et al. Predictors of Resectability and Survival in Patients With Borderline and Locally Advanced Pancreatic Cancer who Underwent Neoadjuvant Treatment With FOLFIRINOX. *Ann Surg.* 2019;269(4):733-740.

11. https://gco.iarc.fr/today/data/factsheets/cancers/13-Pancreas-fact-sheet.pdf. Published 2020. Accessed 05.18.2022.

12. Siegel RL, Miller KD, Fuchs HE, Jemal A. Cancer Statistics, 2021. *CA Cancer J Clin.* 2021;71(1):7-33.

13. Pijnappel EN, Schuurman M, Wagner AD, et al. Sex, Gender and Age Differences in Treatment Allocation and Survival of Patients With Metastatic Pancreatic Cancer: A Nationwide Study. *Front Oncol.* 2022;12:839779.

14. Rawla P, Thandra KC, Sunkara T. Pancreatic cancer and obesity: epidemiology, mechanism, and preventive strategies. *Clin J Gastroenterol.* 2019;12(4):285-291.

15. Eibl G, Rozengurt E. Obesity and Pancreatic Cancer: Insight into Mechanisms. *Cancers (Basel).* 2021;13(20).

16. Michaud DS, Giovannucci E, Willett WC, Colditz GA, Stampfer MJ, Fuchs CS. Physical activity, obesity, height, and the risk of pancreatic cancer. *JAMA.* 2001;286(8):921-929.

17. Li D, Morris JS, Liu J, et al. Body mass index and risk, age of onset, and survival in patients with pancreatic cancer. *JAMA.* 2009;301(24):2553-2562.

18. Di Gioia A, Giuliani T, Marchegiani G, et al. Pancreatoduodenectomy in obese patients: surgery for nonmalignant tumors might be deferred. *HPB (Oxford).* 2021.

19. Mungroop TH, Klompmaker S, Wellner UF, et al. Updated Alternative Fistula Risk Score (ua-FRS) to Include Minimally Invasive Pancreatoduodenectomy: Pan-European Validation. *Ann Surg.* 2021;273(2):334-340.

20. Callery MP, Pratt WB, Kent TS, Chaikof EL, Vollmer CM, Jr. A prospectively validated clinical risk score accurately predicts pancreatic fistula after pancreatoduodenectomy. *J Am Coll Surg.* 2013;216(1):1-14.

21. Hank T, Sandini M, Ferrone CR, et al. Association Between Pancreatic Fistula and Long-term Survival in the Era of Neoadjuvant Chemotherapy. *JAMA Surg.* 2019;154(10):943-951.

22. Tumas J, Tumiene B, Jurkeviciene J, Jasiunas E, Sileikis A. Nutritional and immune impairments and their effects on outcomes in early pancreatic cancer patients undergoing pancreatoduodenectomy. *Clin Nutr.* 2020;39(11):3385-3394.

23. Naumann P, Eberlein J, Farnia B, Hackert T, Debus J, Combs SE. Continued Weight Loss and Sarcopenia Predict Poor Outcomes in Locally Advanced Pancreatic Cancer Treated with Chemoradiation. *Cancers (Basel).* 2019;11(5).

24. Ozola Zalite I, Zykus R, Francisco Gonzalez M, et al. Influence of cachexia and sarcopenia on survival in pancreatic ductal adenocarcinoma: a systematic review. *Pancreatology.* 2015;15(1):19-24.

25. Kanda M, Fujii T, Kodera Y, Nagai S, Takeda S, Nakao A. Nutritional predictors of postoperative outcome in pancreatic cancer. *Br J Surg.* 2011;98(2):268-274.

26. Siegel R, Naishadham D, Jemal A. Cancer statistics, 2012. *CA Cancer J Clin.* 2012;62(1):10-29.

27. Siegel RL, Miller KD, Fuchs HE, Jemal A. Cancer statistics, 2022. *CA Cancer J Clin.* 2022;72(1):7-33.

28. Conroy T, Desseigne F, Ychou M, et al. FOLFIRINOX versus gemcitabine for metastatic pancreatic cancer. *N Engl J Med.* 2011;364(19):1817-1825.

29. Von Hoff DD, Ervin T, Arena FP, et al. Increased survival in pancreatic cancer with nab-paclitaxel plus gemcitabine. *N Engl J Med.* 2013;369(18):1691-1703.

30. Hackert T, Sachsenmaier M, Hinz U, et al. Locally Advanced Pancreatic Cancer: Neoadjuvant Therapy With Folfirinox Results in Resectability in 60% of the Patients. *Ann Surg.* 2016;264(3):457-463.

31. Oettle H, Neuhaus P, Hochhaus A, et al. Adjuvant chemotherapy with gemcitabine and long-term outcomes among patients with resected pancreatic cancer: the CONKO-001 randomized trial. *JAMA.* 2013;310(14):1473-1481.

32. Conroy T, Hammel P, Hebbar M, et al. FOLFIRINOX or Gemcitabine as Adjuvant Therapy for Pancreatic Cancer. *N Engl J Med.* 2018;379(25):2395-2406.

33. Malleo G, Maggino L, Ferrone CR, et al. Does Site Matter? Impact of Tumor Location on Pathologic Characteristics, Recurrence, and Survival of Resected Pancreatic Ductal Adenocarcinoma. *Ann Surg Oncol.* 2020.

34. Dreyer SB, Jamieson NB, Upstill-Goddard R, et al. Defining the molecular pathology of pancreatic body and tail adenocarcinoma. *Br J Surg.* 2018;105(2):e183-e191.

35. Winer LK, Dhar VK, Wima K, et al. The Impact of Tumor Location on Resection and Survival for Pancreatic Ductal Adenocarcinoma. *J Surg Res.* 2019;239:60-66.

36. Bannon SA, Montiel MF, Goldstein JB, et al. High Prevalence of Hereditary Cancer Syndromes and Outcomes in Adults with Early-Onset Pancreatic Cancer. *Cancer Prev Res (Phila).* 2018;11(11):679-686.

37. Ben-Aharon I, Elkabets M, Pelossof R, et al. Genomic Landscape of Pancreatic Adenocarcinoma in Younger versus Older Patients: Does Age Matter? *Clin Cancer Res.* 2019;25(7):2185-2193.

38. Tsang ES, Topham JT, Karasinska JM, et al. Delving into Early-onset Pancreatic Ductal Adenocarcinoma: How Does Age Fit In? *Clin Cancer Res.* 2021;27(1):246-254.

39. He J, Edil BH, Cameron JL, et al. Young patients undergoing resection of pancreatic cancer fare better than their older counterparts. *J Gastrointest Surg.* 2013;17(2):339-344.

40. Ordonez JE, Hester CA, Zhu H, et al. Clinicopathologic Features and Outcomes of Early-Onset Pancreatic Adenocarcinoma in the United States. *Ann Surg Oncol.* 2020;27(6):1997-2006.

41. Burmeister EA, Waterhouse M, Jordan SJ, et al. Determinants of survival and attempted resection in patients with non-metastatic pancreatic cancer: An Australian population-based study. *Pancreatology.* 2016;16(5):873-881.

42. van der Geest LG, Besselink MG, van Gestel YR, et al. Pancreatic cancer surgery in elderly patients: Balancing between short-term harm and long-term benefit. A population-based study in the Netherlands. *Acta Oncol.* 2016;55(3):278-285.

43. Li X, Liu Z, Ye Z, Gou S, Wang C. Impact of age on survival of patients with pancreatic cancer after surgery: Analysis of SEER data. *Pancreatology.* 2018;18(1):133-138.

44. Hank T, Sandini M, Qadan M, et al. Diabetes mellitus is associated with unfavorable pathologic features, increased postoperative mortality, and worse long-term survival in resected pancreatic cancer. *Pancreatology.* 2020;20(1):125-131.

45. Lv X, Qiao W, Leng Y, Wu L, Zhou Y. Impact of diabetes mellitus on clinical outcomes of pancreatic cancer after surgical resection: A systematic review and meta-analysis. *PLoS One.* 2017;12(2):e0171370.

46. Walter U, Kohlert T, Rahbari NN, Weitz J, Welsch T. Impact of preoperative diabetes on long-term survival after curative resection of pancreatic adenocarcinoma: a systematic review and meta-analysis. *Ann Surg Oncol.* 2014;21(4):1082-1089.

47. Hartwig W, Hackert T, Hinz U, et al. Pancreatic cancer surgery in the new millennium: better prediction of outcome. *Ann Surg.* 2011;254(2):311-319.

48. Chaiteerakij R, Petersen GM, Bamlet WR, et al. Metformin Use and Survival of Patients With Pancreatic Cancer: A Cautionary Lesson. *J Clin Oncol.* 2016;34(16):1898-1904.

49. Marchegiani G, Andrianello S, Malleo G, et al. Does Size Matter in Pancreatic Cancer?: Reappraisal of Tumour Dimension as a Predictor of Outcome Beyond the TNM. *Ann Surg.* 2017;266(1):142-148.

50. Watanabe G, Ushida Y, Oba A, et al. Impact of Tumor Size on the Outcomes of Patients with Resectable Distal Pancreatic Cancer: Lessons Learned from a Series of 158 Radical Resections. *Ann Surg Oncol.* 2022;29(1):378-388.

51. Li D, Wang L, Cai W, Liang M, Ma X, Zhao X. Prognostic stratification in patients with pancreatic ductal adenocarcinoma after curative resection based on preoperative pancreatic contrast-enhanced CT findings. *Eur J Radiol.* 2022;151:110313.

52. Raptis DA, Fessas C, Belasyse-Smith P, Kurzawinski TR. Clinical presentation and waiting time targets do not affect prognosis in patients with pancreatic cancer. *Surgeon.* 2010;8(5):239-246.

53. Kelsen DP, Portenoy R, Thaler H, Tao Y, Brennan M. Pain as a predictor of outcome in patients with operable pancreatic carcinoma. *Surgery.* 1997;122(1):53-59.

54. Takikawa T, Kikuta K, Hamada S, et al. Clinical features and prognostic impact of asymptomatic pancreatic cancer. *Sci Rep.* 2022;12(1):4262.

55. Mizuno S, Nakai Y, Isayama H, et al. Diabetes is a useful diagnostic clue to improve the prognosis of pancreatic cancer. *Pancreatology.* 2013;13(3):285-289.

56. Takeda Y, Saiura A, Takahashi Y, et al. Asymptomatic Pancreatic Cancer: Does Incidental Detection Impact Long-Term Outcomes? *J Gastrointest Surg.* 2017;21(8):1287-1295.

57. Hank T, Hinz U, Reiner T, et al. A Pretreatment Prognostic Score to Stratify Survival in Pancreatic Cancer. *Ann Surg.* 2021.

58. Malleo G, Maggino L, Ferrone CR, et al. Reappraising the Concept of Conditional Survival After Pancreatectomy for Ductal Adenocarcinoma: A Bi-institutional Analysis. *Ann Surg.* 2020;271(6):1148-1155.

59. Callery MP, Chang KJ, Fishman EK, Talamonti MS, William Traverso L, Linehan DC. Pretreatment assessment of resectable and borderline resectable pancreatic cancer: expert consensus statement. *Ann Surg Oncol.* 2009;16(7):1727-1733.

60. Al-Hawary MM, Francis IR, Chari ST, et al. Pancreatic ductal adenocarcinoma radiology reporting template: consensus statement of the Society of Abdominal Radiology and the American Pancreatic Association. *Radiology.* 2014;270(1):248-260.

61. Versteijne E, Suker M, Groothuis K, et al. Preoperative Chemoradiotherapy Versus Immediate Surgery for Resectable and Borderline Resectable Pancreatic Cancer: Results of the Dutch Randomized Phase III PREOPANC Trial. *J Clin Oncol.* 2020;38(16):1763-1773.

62. Hartwig W, Werner J, Jager D, Debus J, Buchler MW. Improvement of surgical results for pancreatic cancer. *Lancet Oncol.* 2013;14(11):e476-e485.

63. Maggino L, Malleo G, Marchegiani G, et al. Outcomes of Primary Chemotherapy for Borderline Resectable and Locally Advanced Pancreatic Ductal Adenocarcinoma. *JAMA Surg.* 2019;154(10):932-942.

64. Rangelova E, Wefer A, Persson S, et al. Surgery Improves Survival After Neoadjuvant Therapy for Borderline and Locally Advanced Pancreatic Cancer: A Single Institution Experience. *Ann Surg.* 2021;273(3):579-586.

65. Reni M, Zanon S, Balzano G, et al. Selecting patients for resection after primary chemotherapy for non-metastatic pancreatic adenocarcinoma. *Ann Oncol.* 2017;28(11):2786-2792.

66. https://www.hpbonline.org/article/S1365-182X(21)00645-6/fulltext. Published 2021. Accessed.

67. Ferrone CR, Marchegiani G, Hong TS, et al. Radiological and surgical implications of neoadjuvant treatment with FOLFIRINOX for locally advanced and borderline resectable pancreatic cancer. *Ann Surg.* 2015;261(1):12-17.

68. Versteijne E, Vogel JA, Besselink MG, et al. Meta-analysis comparing upfront surgery with neoadjuvant treatment in patients with resectable or borderline resectable pancreatic cancer. *Br J Surg.* 2018;105(8):946-958.

69. Versteijne E, van Dam JL, Suker M, et al. Neoadjuvant Chemoradiotherapy Versus Upfront Surgery for Resectable and Borderline Resectable Pancreatic Cancer: Long-Term Results of the Dutch Randomized PREOPANC Trial. *J Clin Oncol.* 2022;40(11):1220-1230.

70. Janssen QP, van Dam JL, Bonsing BA, et al. Total neoadjuvant FOLFIRINOX versus neoadjuvant gemcitabine-based chemoradiotherapy and adjuvant gemcitabine for resectable and borderline resectable pancreatic cancer (PREOPANC-2 trial): study protocol for a nationwide multicenter randomized controlled trial. *BMC Cancer.* 2021;21(1):300.

71. Reni M, Balzano G, Zanon S, et al. Safety and efficacy of preoperative or postoperative chemotherapy for resectable pancreatic adenocarcinoma (PACT-15): a randomised, open-label, phase 2-3 trial. *Lancet Gastroenterol Hepatol.* 2018;3(6):413-423.

72. Sohal DPS, Duong M, Ahmad SA, et al. Efficacy of Perioperative Chemotherapy for Resectable Pancreatic Adenocarcinoma: A Phase 2 Randomized Clinical Trial. *JAMA Oncol.* 2021;7(3):421-427.
