# Supplementary material for: Expert Judgment Supporting a Bayesian Network to Model the Survival of Pancreatic Cancer Patients
Source: Cancers (Basel). 2025 Jan 17;17(2):301. doi: 10.3390/cancers17020301 (PMC11764457; doi:10.3390/cancers17020301)
Supplement: Supplementary file 1 [file cancers-17-00301-s001.zip › Supplementary Materials S3.pdf]

Supplementary Materials S3

Experts invitation letter

Dear Colleague,

We would like to invite you to take part in a research project that will not require anything of you other than your opinions, experience, and a brief investment in time. You qualified given your contribution to the field through valuable research, as well as your international standing among peers.

The purpose of this research project is to develop a baseline prediction model identifying causal relationships between clinical characteristics at diagnosis and overall survival following pancreatectomy for pancreatic ductal adenocarcinoma. To do so, we are employing artificial intelligence in the form of a hybrid Bayesian network model. Such an approach compels an elicitation process from pancreatic cancer experts, who will provide their opinions regarding discrete quantities which the statistical model is set upon. To ensure transparency and rigor throughout the process, we decided to employ the Sheffield Elicitation Framework (a.k.a. SHELF), a method which is largely utilized in pharmacological research and industry. Results of this model will be used for the graduation thesis of our Project Manager, Dr. Erica Secchettin (Master Degree in biostatistics), and – possibly – to craft a paper for which we are happy to offer co-authorship. Furthermore, this clinical model will constitute the backbone of a more complex algorithm integrating radiomic and molecular data. Analysis will be carried out under the supervision of Prof. Dario Gregori, Head of Biostatistics at the University of Padua.

In practice, what we are asking you is to express your availability to participate to an online survey through which you will share your judgement regarding the estimated *apriori* contribution of few clinical parameters over 3-year survival following pancreatic resection. Individual opinions will be then fitted in a consensus probability distribution to enter the Bayesian model, which will be initially trained using the pancreatic cancer database at the University of Verona. If necessary, uncertainties and major discrepancies will be solved through a brief Zoom meeting which will be scheduled at your convenience. Please find attached the study proposal and an evidence dossier which you may find useful while preparing to the survey.

We hope you can make this 20–30-minute investment to help us assemble a quality project and are grateful for your consideration and participation. It should be fun and stimulating to do, and an opportunity to share ideas with

colleagues and friends all over the world. Please let us know if you are available by replying to this mail. Thank you!

Sincerely,

The Verona team:

Giuseppe Malleo

Fabio Casciani

Erica Secchettin

Claudio Bassi

Roberto Salvia
