# Supplementary material for: Expert Judgment Supporting a Bayesian Network to Model the Survival of Pancreatic Cancer Patients
Source: Cancers (Basel). 2025 Jan 17;17(2):301. doi: 10.3390/cancers17020301 (PMC11764457; doi:10.3390/cancers17020301)
Supplement: Supplementary file 1 [file cancers-17-00301-s001.zip › Supplementary Materials S4.pdf]

**Supplementary Materials S4**

*Survey administered to experts at first step of workshop.*

Expert Elicitation Process
As an expert in the field of pancreatic cancer, we are asking you to answer the following questions regarding the perceived contribution of distinct baseline clinical characteristics to the prognosis of patients undergoing pancreatectomy for PDAC. At your convenience, you can refer to an evidence dossier (see email attachment) reviewing updated evidence regarding each of the elements explored in the present questionnaire.

**Variable #1: Ca19.9**
***Description*: baseline Ca 19.9 serum level (IU/mL). In case of jaundice, consider values following biliary drainage**
***Variable distribution*: continuous**
What would be, in your opinion, the **baseline Ca19.9 cutoff** below which you are reasonably confident that a patient will be alive 36 months after pancreatectomy?
Given this cutoff, could you provide a **lower bound** of confidence (numeric value) based on your gut feeling?
Given this cutoff, could you provide an **upper bound** of confidence (numeric value) based on your gut feeling?

**Variable #1_bis: Ca19.9 variation after neoadjuvant treatment**
***Description*: Ca19.9 percentage decrease relative to baseline value (delta)**
***Variable distribution*: continuous**

What would be, in your opinion, the **delta Ca19.9** above which you are reasonably confident that the patient will be alive 36 months after post-neoadjuvant pancreatectomy?
Given this delta, could you provide a **lower bound** of confidence based on your gut feeling?
Given this delta, could you provide an **upper bound** of confidence based on your gut feeling?

**Variable #2: Gender**
***Description*: patient gender**
***Variable distribution*: dichotomous (male vs. female)**
Which variable level, in your opinion, is associated with a **greater chance** of being alive 36 months after pancreatectomy?
○ Male (patients alive at 36 months have a probability > 50% to be men)
○ Female (patients alive at 36 months have a probability > 50% to be women)
○ None (among patients alive at 36 months, 50% are men and 50% are women)
Considering the variable level you have selected (male or female), what is in your view the **positive deviation** of survival probability from the middle point (50%/50%)?

**Example:** selecting "female" and assuming a positive deviation of 15% indicates a probability of (50+15) = 65% that a patient alive at 36 months is female, as opposed to the probability of being male of (50-15) = 35%.
**If you selected "none" this value will be 0**

Could you specify a **lower bound** of confidence around this probability deviation (numeric value) based on your gut feeling?
Could you specify an **upper bound** of confidence around this probability deviation (numeric value) based on your gut feeling?
**Variable #3: Body Mass Index**
***Description*: Body Mass Index (BMI), calculated as patient weight at diagnosis divided by square height**
***Variable distribution*: dichotomous (normal/overweight [BMI ≤30] vs. obese [BMI >30])**
Which variable level, in your opinion, is associated with a **greater chance** of being alive 36 months after pancreatectomy?
○ Normal/overweight (patients alive at 36 months have a probability > 50% of being normal/overweight at the time of diagnosis)
○ Obesity (patients alive at 36 months have a probability > 50% of being obese at the time of diagnosis)
○ None (among patients alive at 36 months, 50% were normal/overweight and 50% were obese at the time of diagnosis)

Considering the variable level you have selected (normal/overweight or obese), what is in your view the **positive deviation** of survival probability from the middle point (50%/50%)?
**Example:** selecting "normal/overweight" and assuming a positive deviation of 15% indicates a probability of (50+15) = 65% that a patient alive at 36 months is normal/overweight, as opposed to the probability to be obese of (50-15) = 35%.
**If you selected "none" this value will be 0**
Could you specify a **lower bound** of confidence around this probability deviation (numeric value) based on your gut feeling?
Could you specify an **upper bound** of confidence around this probability deviation (numeric value) based on your gut feeling?
**Variable #4: Year of diagnosis**
***Description*: year of first diagnosis**
***Variable distribution*: dichotomous (before Dec 31st 2014 vs. after Jan 1st 2015, full adoption of neoadjuvant multiagent regimens, gemcitabine + nab-paclitaxel/FOLFIRINOX)**
Which variable level, in your opinion, is associated with a **greater chance** of being alive 36 months after pancreatectomy?
○ Before Dec 31st 2014 (patients alive at 36 months have a probability > 50% of having been diagnosed before Dec 31st 2014)
○ After Jan 1st 2015 (patients alive at 36 months have a probability > 50% of having been diagnosed after Jan 1st 2015)
○ None (among patients alive at 36 months, 50% had been diagnosed before 2015 and 50% after 2015)

Considering the variable level you have selected (before or after 2015), what is in your view the **positive deviation** of survival probability from to the middle point (50%/50%)?
**Example:** selecting "after 2015" and assuming a positive deviation of 15% indicates a probability of (50+15) = 65% that a patient alive at 36 months has received pancreatectomy from 2015, as opposed to the probability of receiving pancreatectomy before 2015 of (50-15) = 35%.
**If you selected "none" this value will be 0**
Could you specify a **lower bound** of confidence around this probability deviation (numeric value) based on your gut feeling?
Could you specify an **upper bound** of confidence around this probability deviation (numeric value) based on your gut feeling?

**Variable #5: Tumor location**
***Description*: tumor location**
***Variable distribution*: dichotomous (‘head’ [also including uncinate process and neck] vs. ‘body-tail’)**
Which variable level, in your opinion, is associated with a **greater chance** of being alive 36 months after pancreatectomy?
○ Head (patients alive at 36 months have a probability > 50% of having head PDAC)
○ Body-tail (patients alive at 36 months have a probability > 50% of having body-tail PDAC)
○ None (among patients alive at 36 months, 50% had head PDAC and 50% body-tail PDAC)

Considering the variable level you have selected (head vs. body-tail), what is in your view the **positive deviation** of survival probability from the middle point (50%/50%)?
**Example:** selecting "head" and assuming a positive deviation of 15% indicates a probability of (50+15) = 65% that a patient alive at 36 months had a head tumor, as opposed to the probability of having had a body-tail tumor of (50-15) = 35%.
**If you selected "none" this value will be 0**
Could you specify a **lower bound** of confidence around this probability deviation (numeric value) based on your gut feeling?
Could you specify an **upper bound** of confidence around this probability deviation (numeric value) based on your gut feeling?
**Variable #6: Age**
***Description*: patient age (expressed as round years) at the time of diagnosis**
***Variable distribution*: continuous**
What would be, in your opinion, the **age cutoff** below which you are reasonably confident that the patient will be alive 36 months after pancreatectomy?
Given this cutoff, can you provide a **lower bound** of confidence (numeric value) based on your gut feeling?
Given this cutoff, can you provide an **upper bound** of confidence (numeric value) based on your gut feeling?
**Variable #7: Diabetes**
***Description*: presence of diabetes at the time of diagnosis**
***Variable distribution*: dichotomous (yes vs. no)**

Which variable level, in your opinion, is associated with a **greater chance** of being alive 36 months after pancreatectomy?
o Presence of diabetes (patients alive at 36 months have a probability > 50% of being diabetic at the time of diagnosis)
o Absence of diabetes (patients alive at 36 months have a probability > 50% of being non-diabetic at the time of diagnosis)
o None (among patients alive at 36 months, 50% were diabetic and 50% non-diabetic)

Considering the variable level you have selected (diabetes yes vs. no), what is in your view the **positive deviation** of survival probability from the middle point (50%/50%)?
**Example:** selecting "absence of diabetes" and assuming a positive deviation of 15% indicates a probability of (50+15) = 65% that a patient alive at 36 months was not diabetic, as opposed to the probability of having been diabetic of (50-15) = 35%.
**If you selected "none" this value will be 0**
Could you specify a **lower bound** of confidence around this probability deviation (numeric value) based on your gut feeling?
Could you specify an **upper bound** of confidence around this probability deviation (numeric value) based on your gut feeling?
**Variable #8: Tumor size**
***Description*: greatest axial dimension (in millimeters) at preoperative imaging (baseline in upfront resection, post-treatment in patients receiving neoadjuvant therapy)**
***Variable distribution*: continuous**
What would be, in your opinion, the **tumor size cutoff** below which you are reasonably confident that the patient will be alive 36 months after pancreatectomy?
Given this cutoff, can you provide a **lower bound** of confidence (numeric value) based on your gut feeling?
Given this cutoff, can you provide an **upper bound** of confidence (numeric value) based on your gut feeling?
**Variable #9: Presence of symptoms**
***Description*: presence of any of the following at the time of diagnosis: jaundice, significant weight loss (≥10% in the last six months), gastric outlet obstruction, epigastric or back pain**
***Variable distribution*: dichotomous (yes vs. no)**
Which variable level, in your opinion, is associated with a **greater chance** of being alive 36 months after pancreatectomy?
o Presence of symptoms (patients alive at 36 months have a probability > 50% of having been symptomatic at the time of diagnosis)
o Absence of symptoms (patients alive at 36 months have a probability > 50% of being asymptomatic at the time of diagnosis)
o None (among patients alive at 36 months, 50% were symptomatic and 50% were asymptomatic at the time of diagnosis)

Considering the variable level you have selected (symptoms vs. no symptoms), what is in your view the **positive deviation** of survival probability from the middle point (50%/50%)?

**Example:** selecting "no symptoms" and assuming a positive deviation of 15% indicates a probability of (50+15) = 65% that a patient alive at 36 months was not symptomatic, as opposed to the probability of having been symptomatic of (50-15) = 35%.
**If you selected "none" this value will be 0**
Could you specify a **lower bound** of confidence around this probability deviation (numeric value) based on your gut feeling?
Could you specify an **upper bound** of confidence around this probability deviation (numeric value) based on your gut feeling?
**Variable #10: American Association of Anesthesiology (ASA) Score**
***Description*: preoperative ASA score**
***Variable distribution*: dichotomous (ASA I-II vs. ASA III-IV)**
Which variable level, in your opinion, is associated with a **greater chance** of being alive 36 months after pancreatectomy?
○ ASA status I-II (patients alive at 36 months have a probability > 50% of having been ASA I-II preoperatively)
○ ASA status III-IV (patients alive at 36 months have a probability > 50% of having been ASA III-IV preoperatively)
○ None (among patients alive at 36 months, 50% were ASA I-II and 50% were ASA III-IV preoperatively)

Considering the variable level you have selected (ASA I-II vs. III-IV), what is in your view the **positive deviation** of survival probability from the middle point (50%/50%)?
**Example:** by selecting "ASA I-II" and assuming a positive deviation of 15% indicates a probability of (50+15) = 65% that a patient alive at 36 months was ASA score I-II, as opposed to the probability of having been ASA score III-IV of (50-15) = 35%.
**If you selected "none" this value will be 0**
Could you specify a **lower bound** of confidence around this probability deviation (numeric value) based on your gut feeling?
Could you specify an **upper bound** of confidence around this probability deviation (numeric value) based on your gut feeling?
**Variable #11: Resectability status**
***Description*: anatomical resectability at the time of diagnosis, defined per the National Comprehensive Cancer Network (NCCN) criteria**
***Variable distribution*: dichotomous (resectable vs. borderline resectable/locally advanced)**
Which variable level, in your opinion, is associated with a **greater chance** of being alive 36 months after pancreatectomy?
○ Resectable tumor (patients alive at 36 months have a probability > 50% of having had a resectable tumor)
○ Borderline resectable/locally advanced tumor (patients alive at 36 months have a probability > 50% of having had borderline resectable/locally advanced tumor)
○ None (among patients alive at 36 months, 50% had resectable tumors and 50% had borderline resectable/locally advanced tumors)

Considering the variable level you have selected (resectable vs. borderline/locally advanced), what is in your view the **positive deviation** of survival probability from the middle point (50%/50%)?
**Example:** selecting "resectable" and assuming a positive deviation of 15% indicates a probability of (50+15) = 65% that a patient alive at 36 months had a resectable tumor, as opposed to the probability of having had a borderline resectable/locally advanced tumor (50-15) = 35%.
**If you selected "none" this value will be 0**
Could you specify a **lower bound** of confidence around this probability deviation (numeric value) based on your gut feeling?
Could you specify an **upper bound** of confidence around this probability deviation (numeric value) based on your gut feeling?
**Variable #11_bis: Presence of any vascular involvement at diagnosis**
***Description*: presence of any arterial or venous solid tumor contact at baseline imaging**
***Variable distribution*: dichotomous (yes vs. no)**
Which variable level, in your opinion, is associated with a **greater chance** of being alive 36 months after pancreatectomy?

- No vascular involvement (patients alive at 36 months have a probability > 50% of having had a tumor without any vascular involvement)
- Any vascular involvement (patients alive at 36 months have a probability > 50% of having had a tumor with any degree of vascular involvement)
- None (among patients alive at 36 months, 50% had tumors without vascular involvement and 50% had tumors with any degree of vascular involvement)

Considering the variable level you have selected (no vascular involvement vs. any vascular involvement), what is in your view the **positive deviation** of survival probability from the middle point (50%/50%)?
**Example:** selecting "no vascular involvement" and assuming a positive deviation of 15% indicates a probability of (50+15) = 65% that a patient alive at 36 months had a PDAC without vascular involvement, as opposed to the probability of having had a PDAC with some vascular involvement (50-15) = 35%.
**If you selected "none" this value will be 0**
Could you specify a **lower bound** of confidence around this probability deviation (numeric value) based on your gut feeling?
Could you specify an **upper bound** of confidence around this probability deviation (numeric value) based on your gut feeling?
**Variable #12: Neoadjuvant treatment**
***Description*: completion of at least 3 months of preoperative chemotherapy, with or without radiation therapy**
***Variable distribution*: dichotomous (yes vs no)**
Which variable level, in your opinion, is associated with a **greater chance** of being alive 36 months after pancreatectomy?

- Neoadjuvant treatment (patients alive at 36 months have a probability > 50% of having received neoadjuvant treatment)
- Upfront resection (patients alive at 36 months have a probability > 50% of not having received neoadjuvant therapy)
- None (among patients alive at 36 months, 50% had received neoadjuvant treatment and 50% had received upfront resection)

Considering the variable level you have selected (neoadjuvant therapy vs. upfront resection), what is in your view the **positive deviation** of survival probability from the middle point (50%/50%)?
**Example:** selecting "neoadjuvant therapy" and assuming a positive deviation of 15% indicates a probability of (50+15) = 65% that a patient alive at 36 months had received neoadjuvant therapy, as opposed to the probability of having received upfront surgery (50-15) = 35%.
**If you selected "none" this value will be 0**
Could you specify a **lower bound** of confidence around this probability deviation (numeric value) based on your gut feeling?
Could you specify an **upper bound** of confidence around this probability deviation (numeric value) based on your gut feeling?
