# Supplementary material for: Expert Judgment Supporting a Bayesian Network to Model the Survival of Pancreatic Cancer Patients
Source: Cancers (Basel). 2025 Jan 17;17(2):301. doi: 10.3390/cancers17020301 (PMC11764457; doi:10.3390/cancers17020301)
Supplement: Supplementary file 1 [file cancers-17-00301-s001.zip › Supplementary Materials S5.pdf]

Formula (S1).

$$estBetaParams < -funcion(\mu, \quad \sigma)\{$$

$$\alpha < -(\frac{1-\mu}{\sigma - 1/\mu)} * \ \mu^2$$

$$\beta < -\alpha * \left(\frac{1}{\mu} - 1\right)$$

$$return\left(params = list(alpha = \alpha, beta = \beta)\right)\}$$
