# Supplementary material for: Expert Judgment Supporting a Bayesian Network to Model the Survival of Pancreatic Cancer Patients
Source: Cancers (Basel). 2025 Jan 17;17(2):301. doi: 10.3390/cancers17020301 (PMC11764457; doi:10.3390/cancers17020301)
Supplement: Supplementary file 1 [file cancers-17-00301-s001.zip › Supplementary Materials S6.pdf]

Supplementary Materials S6 – Technical Appendix

From Elicited Marginal to Conditional Probabilities

Imagine we're working with a simplified BN designed to predict the likelihood of long-term survival in pancreatic cancer patients. Our network includes a few key predictor variables:

1. **Tumor Size** – This could be categorized simply as small, medium, or large, even though it's originally continuous.
2. **CA 19-9 Levels** – This biomarker might be categorized as low, medium, or high.
3. **Survival Outcome** – This is the outcome we're interested in, defined as long-term or short-term survival.

In the BN, we start with **marginal probabilities** for each variable. Marginal probabilities give us the likelihood of different values for each variable based on prior expert judgment without considering any specific relationships with other variables.

For instance, let's say that, from prior data or expert estimates:

- The probability of a medium tumor size (P(Tumor Size = medium)) is 0.5, meaning that about 50% of pancreatic cancer patients are likely to have tumors of this size.
- The probability of high CA 19-9 levels (P(CA 19-9 Levels = high)) is 0.3, indicating that 30% of patients are expected to show high levels of this biomarker.
- The probability of long-term survival (P(Survival Outcome = long-term)) is 0.4, suggesting that, on average, 40% of patients experience long-term survival.

However, these marginal probabilities alone aren't sufficient for making personalized predictions because they don't account for the relationships between variables. This is where **conditional probabilities** come in. Conditional probabilities help us understand the likelihood of one event, given that we know something about another. For example, we might look at the probability of long-term survival specifically for patients with a medium-sized tumor and high CA 19-9 levels.

We can calculate these conditional probabilities by using **observed data** from patients with known outcomes. Suppose that, in the dataset, we find that among those with medium-sized tumors and high CA 19-9 levels, only 25% survived long-term. We'd express this relationship as **P(Survival Outcome = long-term | Tumor Size = medium, CA 19-9 Levels = high) = 0.25**. In other words, patients with this specific profile have a 25% chance of long-term survival.

Now, let's combine this conditional probability with the marginal probabilities to calculate the overall likelihood of a patient with these characteristics. First, we estimate the **joint probability** of having both a medium tumor size and high CA 19-9 levels. If we know that 60% of patients with a medium tumor also have high CA 19-9 levels, we can calculate this as:

$$P(\text{ Tumor Size } = \text{ medium, CA 19-9 Levels } = \text{ high }) = P(\text{ Tumor Size } = \text{ medium }) \times P(\text{ CA 19-9 Levels } = \text{ high } \mid \text{ Tumor Size } = \text{ medium })$$

Using our values, this becomes:

$$P(\text{ Tumor Size } = \text{ medium, CA 19-9 Levels } = \text{ high }) = 0.5 \times 0.6 = 0.3$$

Finally, we can use **Bayes' theorem** to find the probability of long-term survival given this profile. Bayes' theorem combines prior information with observed data to update our understanding based on new evidence. Here's how we'd set it up:

$$P(\text{ Survival Outcome } = \text{ long-term } \mid \text{ Tumor Size } = \text{ medium, CA 19-9 Levels } = \text{ high }) = \frac{P(\text{ Survival Outcome } = \text{ long-term }) \times P(\text{ Tumor Size } = \text{ medium, CA 19-9 Levels } = \text{ high } \mid \text{ Survival Outcome } = \text{ long-term })}{P(\text{ Tumor Size } = \text{ medium, CA 19-9 Levels } = \text{ high })}$$

Using our numbers:

- P(Survival Outcome = long-term) = 0.4
- P(Tumor Size = medium, CA 19-9 Levels = high | Survival Outcome = long-term) = 0.25 (from the observed data)
- P(Tumor Size = medium, CA 19-9 Levels = high) = 0.3

Plugging these values into Bayes' theorem gives us:

$$P(\text{ Survival Outcome } = \text{ long-term } \mid \text{ Tumor Size } = \text{ medium, CA 19-9 Levels } = \text{ high }) = \frac{0.4 \times 0.25}{0.3} = \frac{0.1}{0.3} \approx 0.33$$

This calculation tells us that, given a patient with a medium-sized tumor and high CA 19-9 levels, there's about a 33% chance of long-term survival. This result combines our prior knowledge and patient data, providing a personalized probability tailored to the patient's specific characteristics.

In a Bayesian Network, these calculations happen across all nodes, with each variable updating its probability about others. This dynamic updating allows the BN to provide highly customized predictions based on the patient's unique clinical profile.

Obtaining Posterior via Markov Chain Monte Carlo (MCMC) resampling

To explain the application, we report the example using exact probability values. In the BN model application, we must quantify the uncertainty in a posterior distribution. Exact analytical solutions may be complex or infeasible to derive a posterior distribution; for this reason, we use MCMC to sample from these distributions. MCMC allows us to generate a sequence of random samples from the joint distribution of the BN, which captures the probability of each node given its dependencies.

With MCMC, we iteratively sample from the distribution of each variable, conditioned on the values of its parent nodes in the network. For example:

- We sample from P(Tumor Size | CA 19-9 Levels) and P(Survival Outcome | Tumor Size, CA 19-9 Levels) in a way that respects their relationships.

- Over many iterations, MCMC generates a distribution of possible values for each variable, gradually refining these distributions by resampling based on observed values.

As we observe new data (e.g., a patient's specific tumor size and CA 19-9 levels), the MCMC process updates our posterior distributions by resampling based on the new evidence. Bayes' theorem underlies this update, but MCMC efficiently handles the calculations by simulating distributions rather than calculating them directly. In our example, after observing a patient with a medium tumor and high CA 19-9 levels, MCMC resampling would yield an updated probability distribution for long-term survival specific to this patient profile.

Once MCMC has converged, we obtain stable, refined distributions for each variable.

Sensitivity Analysis Plan

<u>Prior distribution</u>

The sensitivity analysis will systematically test the robustness of the Bayesian Network (BN) by perturbing the priors, conditional probabilities, and DAG structure. We will use the following configurations behind expert-derived prior distributions for the priors:

- Uninformative Priors. Uniform priors will be applied across all variables to simulate a scenario without prior information. For instance, for CA 19-9 levels, the uniform prior will span the plausible clinical range identified from the literature. At the same time, categorical variables like ASA scores will have equal probabilities for all categories.

- Empirical Priors. Marginal probabilities will be estimated directly from available datasets. For continuous variables such as tumor size, these priors will be modeled using distributions (e.g., normal, gamma) fitted to the empirical data. For categorical variables, empirical probabilities will reflect the observed frequencies in the dataset.

The impact of these priors will be assessed by recomputing posterior distributions and evaluating predictive performance.

<u>DAG structure</u>

To validate the DAG structure, we will compare the expert-driven DAG with a data-driven DAG generated using a structural learning algorithm, such as the PC algorithm or Hill-Climbing, applied to the dataset containing the selected clinical variables. The data-driven DAG will serve as a baseline for identifying potential discrepancies or overlooked relationships, such as indirect effects or conditional dependencies not captured in the expert-driven DAG. Both structures will be evaluated using information-theoretic measures, such as the Bayesian Information Criterion (BIC), to assess model fit and complexity.

The estimated models will be compared against existing classical benchmarks like Cox regression.

Validation Metrics

Metrics such as the Brier score, logarithmic score, and area under the receiver operating characteristic (ROC) curve will be calculated to compare model outputs under different prior configurations. Calibration plots will also be used to assess the alignment of predictions with observed data.
